# Azithromycin represses evolution of ceftazidime/avibactam resistance by translational repression of *rpoS* in *Pseudomonas aeruginosa*

**DOI:** 10.1128/jb.00552-24

**Published:** 2025-04-30

**Authors:** Congjuan Xu, Jie Feng, Yuchen Zhou, Huan Ren, Xiaolei Pan, Shuiping Chen, Xuehua Liu, Guanxian Li, Jinjin Li, Bin Geng, Linlin Gao, Zhihui Cheng, Yongxin Jin, Un-Hwan Ha, Shouguang Jin, Iain L. Lamont, Daniel Pletzer, Weihui Wu

**Affiliations:** 1 State Key Laboratory of Medicinal Chemical Biology, Key Laboratory of Molecular Microbiology and Technology of the Ministry of Education, Department of Microbiology, College of Life Sciences, Nankai University, Tianjin, China; 2 Department of Infection and Control, 5th Medical Center of PLA General Hospital, Beijing, China; 3 Tianjin Union Medical Center, Nankai University Affiliated Hospital 535512, Tianjin, China; 4 Department of Biotechnology and Bioinformatics, Korea University, Sejong, South Korea; 5 Department of Biochemistry, University of Otago 2495, Dunedin, Otago, New Zealand; 6 Department of Microbiology and Immunology, University of Otago 626306, Dunedin, New Zealand; Geisel School of Medicine at Dartmouth, Hanover, New Hampshire

**Keywords:** *Pseudomonas aeruginosa*, antibiotic combination, ceftazidime-avibactam

## Abstract

Antibiotic combinations can slow down resistance development and/or achieve synergistic therapeutic effects. In this study, we observed that a combined use of ceftazidime-avibactam (CZA) with azithromycin effectively repressed CZA resistance development in *Pseudomonas aeruginosa*. Transcriptome analysis revealed that subinhibitory concentrations of azithromycin reduced the expression of genes involved in stress-induced mutagenesis, including the stress response sigma factor *rpoS*. Interestingly, ribosome profiling revealed global redistribution of ribosomes by azithromycin, among which ribosome stalling was significantly intensified near the 5´ terminus of the *rpoS* mRNA. Further DNA mutational analysis revealed that azithromycin represses the translation of *rpoS* through its 5´-terminal rare codons, which in turn reduced its transcription. These *in vitro* observations have been recapitulated *in vivo* where azithromycin-repressed CZA resistance development when *P. aeruginosa* was passaged in mice. Overall, our study revealed the molecular mechanism of azithromycin-mediated repression of antibiotic resistance development, providing a promising antibiotic combination for the treatment of *P. aeruginosa* infections.

**IMPORTANCE:**

Antibiotic resistance, a global public health challenge, demands the development of novel antibiotics and therapeutic strategies. Ceftazidime-avibactam (CZA) is a combination of a β-lactam antibiotic with a β-lactamase inhibitor that is effective against various gram-negative bacteria such as *Pseudomonas aeruginosa*. However, clinical CZA-resistant isolates have been reported. Here, we found that combining CZA with azithromycin can effectively suppress the development of resistance in *P. aeruginosa in vitro* and *in vivo*. Moreover, we found that azithromycin represses the translation initiation of *rpoS* through its 5´-terminal rare and less frequent codons, thereby subsequently reducing the mutational frequency of CZA resistance. Therefore, our work provides a promising antibiotic combination for the treatment of *P. aeruginosa* infections.

## INTRODUCTION

The rising antibiotic resistance crisis imposes a severe threat to human health (1). In 2024, the WHO updated its list of antibiotic-resistant bacteria that urgently require new effective treatments. Notably, fluoroquinolone-resistant *Salmonella* Typhi and *Shigella* spp., along with carbapenem-resistant *Pseudomonas aeruginosa*, were listed in the high priority category (2).

*P. aeruginosa* is intrinsically highly resistant to a variety of antibiotics. The resistance mechanisms include low outer membrane permeability, multidrug efflux pumps, and antibiotic-inactivating enzymes (3). Susceptibility to β-lactams can be further reduced by mutations that reduce antibiotic entry into the bacteria, increase efflux, modify target penicillin-binding proteins, or upregulate expression of AmpC β-lactamase (4).

Combination of β-lactamase inhibitors with β-lactams represents a major strategy against β-lactamase-mediated resistance (5). Avibactam is a non-β-lactam β-lactamase inhibitor that can efficiently inhibit the activities of a range of β-lactamases (6). The ceftazidime-avibactam (CZA) combination was approved by the FDA in 2015 for the treatment of ventilator-associated pneumonia and complicated urinary tract and intra-abdominal infections (7). *P. aeruginosa* clinical isolates collected from 70 US medical centers from 2017 to 2018 exhibited a 96.0% susceptibility rate to CZA (8). More recently, CZA displayed better *in vitro* efficacy than imipenem/relebactam and meropenem/vaborbactam against carbapenemase-producing *P. aeruginosa* clinical isolates (9). The development of CZA-resistant *P. aeruginosa* clones was documented during the course of treatment of a patient with CZA in the USA due to a gain-of-function mutation in the *ampC* gene (10). The same mutation was identified in CZA-resistant *P. aeruginosa* clones that developed within a patient in France (11).

Antibiotic combination therapy has been used as a strategy to treat infections and combat resistance evolution (12). For instance, the combination of colistin and tobramycin has been shown to effectively target various species of biofilm-associated bacteria, thus enhancing eradication rates (13). Recently, a study demonstrated that polymyxin B (PMB) combined with cefepime-avibactam effectively eradicates biofilms of PMB-resistant *P. aeruginosa*, both *in vitro* and *in vivo* (14). Additionally, antibiotics like azithromycin (AZM) have been shown to disrupt quorum sensing (QS) pathways and reduce alginate production, thereby interfering with biofilm formation and bacterial virulence (15, 16). To identify antibiotic combinations that repress the evolution of CZA resistance, we combined CZA with various antibiotics, including aztreonam, amikacin, azithromycin, fosfomycin, and ciprofloxacin, and examined the resistance development of a *P. aeruginosa* reference strain PA14 and a previously characterized carbapenem-resistant clinical isolate CI-PA41 (17). We found that azithromycin represses resistance evolution *in vitro* and *in vivo*. Azithromycin reduced the mutation frequency through translational repression of the *rpoS* gene that encodes an alternative sigma factor.

## MATERIALS AND METHODS

### Bacterial strains and plasmids

Bacterial strains, plasmids, and primers used in the study are listed in Table S1. Bacteria were cultured in Luria-Bertani (LB) medium (1% tryptone, 0.5% yeast extract, 1% NaCl, pH=7.4) under aerobic conditions at 37°C.

### Antimicrobial susceptibility test

The minimum inhibitory concentrations (MICs) were determined using the twofold serial dilution method following Clinical and Laboratory Standards Institute guidelines in CAMHB (cation-adjusted Mueller-Hinton broth). Avibactam was used at a fixed concentration of 4 mg/L in combination with ceftazidime (18).

### Experimental evolution assay

Experimental *in vitro* evolution assay was performed as previously described (19). Overnight cultures of *P. aeruginosa* strains were diluted 1:100 in fresh CAMHB with increasing concentrations of CZA in combination with other antibiotics. After 24 hours, cells from the highest concentration of combined antibiotics that allowed growth to a minimum OD_600_ of 2.0 were inoculated into fresh CAMHB with increased concentrations of combined antibiotics for another round of passage. Meanwhile, a portion of the bacteria was inoculated into CAMHB for MIC determination. The passage was repeated for 8 or 10 times, and single colonies were obtained from the replicates by streaking and then used for MIC determination. Antibiotic concentrations used in the *in vitro* passaging experiment are listed in Table S2 to S4.

### Mutation frequency assessment

The mutation frequency was determined following a previously described method with minor modifications (20). Overnight bacterial culture was diluted into 3 mL LB to achieve a final concentration of 10^5^ colony forming units (CFU)/mL, in the absence or presence of 1 mg/L CZA (1/2 MIC) or in combination with various concentrations of azithromycin (the experiment of Fig. 2). In another assay (the experiment of Fig. 3C and 6B), the bacteria were cultured in 3 mL LB to achieve a final concentration of 10^7^ CFU/mL, in the absence or presence of 3 mg/L CZA or in combination with various concentrations of azithromycin. The bacteria were grown for 6 hours, then washed with fresh LB and allowed to recover for 20 hours in LB. The total CFUs were determined on LB agar plates, and the numbers of CZA-resistant mutants were determined by plating the appropriate dilutions on LB plates containing 16 mg/L CZA.

### Bacterial survival assay

Overnight bacterial culture was diluted into 30 mL LB to achieve a final concentration of 10^7^ CFU/mL and then grown with or without antibiotic treatment. At indicated time points, 1 mL of the bacteria was collected and washed with normal saline (0.9% NaCl). The number of viable bacteria was determined by plating.

### Total RNA isolation and quantitative real-time polymerase chain reaction (PCR)

Overnight bacterial cultures were diluted 1:100 into fresh medium with or without indicated antibiotics. After 2.5 hours, bacteria grown in LB or with CZA (3 mg/L) reached the OD_600_ of 1.0. The bacteria were collected by centrifugation and stored at −80°C. Then, 0.5 hours later, bacteria grown with AZM (16 mg/L) and with the combination of CZA (3 mg/L) and AZM (16 mg/L) reached the OD_600_ of 1.0. The bacteria were collected and frozen at −80°C. Total RNA was extracted using the RNAprep Pure Cell/Bacteria Kit (Tiangen Biotech, Beijing, China). For quantitative real-time PCR, the extracted RNA was reverse transcribed into cDNA using random primers and PrimeScript Reverse Transcriptase (Vazyme, Nanjing, China). The resulting cDNA was mixed with specified primers (Table S1) and SYBR premix Ex Taq II (Vazyme, Nanjing, China) for amplification in a Fluorescent Quantitative PCR Detection System (Bioer, Hangzhou, China). The *rpsL* gene that encodes the 30S ribosomal protein was used as the internal control.

### Transcriptome sequencing

RNA sequencing and analysis were conducted by GENEWIZ (Suzhou, China). RNA-Seq was conducted with two biological replicates of each sample. Briefly, the quality of total RNAs was assessed using Agilent Bioanalyzer 2100 (Agilent Technologies, Palo Alto, CA, USA), NanoDrop (Thermo Fisher Scientific Inc.), and 1% agarose gel, and the quantity was determined using Qubit 2.0 Fluorometer (Invitrogen, Carlsbad, CA, USA). For library preparation, rRNA was removed from the total RNA using a specialized rRNA removal kit (GENEWIZ, Suzhou, China). Next, cDNA was synthesized from 1 µg of total RNA. Adaptors were added, and PCR amplification was performed. The libraries were sequenced on Illumina HiSeq/NovaSeq instrument according to manufacturer’s instructions (Illumina, San Diego, CA, USA) with a 2 × 150 bp read length. The average sequencing depth was approximately 2 gigabases per sample. Image analysis and base calling were carried out using HiSeq Control Software + OLB + GAPipeline-1.6 for HiSeq, NovaSeq Control Software + OLB + GAPipeline-1.6 for NovaSeq, and Zebeacall for MGI2000.

### Transcriptome data analysis

Quality control was performed to remove technical sequences, including adapters, PCR primers, or fragments thereof, and bases with quality scores lower than 20. The filtered FASTQ-format data were processed using Cutadapt (version 1.9.1) with a Phred cutoff of 20, an error rate of 0.1, adapter overlap of 1 bp, a minimum length of 75, and a proportion of N of 0.1. The reference genome (PA14) sequence was indexed using Bowtie2 (v.2.2.6), and clean data were aligned to the reference genome using Bowtie2 (v.2.2.6). Expression analysis began with converting transcripts in the fasta format from known gff annotation files. Using this file as the reference gene file, HTSeq (v.0.6.1p1) was used to estimate gene expression levels from the paired-end clean data. Differential expression analysis was conducted using the DESeq2 Bioconductor package, which is based on a negative binomial distribution model. Genes with an adjusted *P*-value of less than 0.05, after adjusting for the false discovery rate using the Benjamini and Hochberg approach, were considered significantly differentially expressed. Gene Ontology (GO) enrichment analysis was performed using GOSeq (v.1.34.1) to identify GO terms that annotate the list of enriched genes with a significant *P*-value of less than 0.05, and topGO was used to plot the directed acyclic graph.

### Western blot analysis

Protein samples from equivalent number of bacterial cells were separated on a 12% SDS-PAGE. The proteins were then transferred onto a PVDF (polyvinylidene difluoride) membrane (Millipore, USA). For protein detection, the PVDF membrane was probed with mouse monoclonal antibodies against 6×His tag (Cell Signaling Technology, USA) and the RNA polymerase alpha subunit RpoA (BioLegend). Signal visualization was performed using the Immobilon Western kit (Millipore) and detected by a Bio-Rad imager (ChemiDocXRS).

### Ribosome profiling

Ribosome footprinting was conducted by CloudSeq Biotech Inc. (Shanghai, China). An overnight culture of wild-type PA14 was diluted 1:100 into fresh medium with or without 16 mg/L azithromycin until late logarithmic phase (OD _600_ ~1). The cells were harvested by centrifugation at 12,000 rpm for 2 minutes at 4°C and immediately flash-frozen in liquid nitrogen. Upon thawing, the cells underwent cycloheximide treatment, followed by lysis and nuclease digestion. The digested samples were used to isolate individual ribosomes using a chromatographic column. The protected RNA fragments were isolated through polyacrylamide gel electrophoresis and treated with rRNA depletion reagents. The resulting RNA was end-repaired, ligated with a 3´ adapter, and reverse-transcribed into cDNA. The cDNA was further purified and PCR-amplified. High-throughput sequencing was performed on an Illumina NovaSeq 6000 platform. The raw data were quality-controlled by Q30. After 3´ adaptor-trimming and low-quality reads removal by Cutadapt (v.1.9.3), the high-quality clean reads were aligned to the reference genome with Tophat2. Then, HTSeq (v.0.9.1) was used to get the raw count, and edgeR was used to perform normalization. Differentially expressed mRNAs were identified by *P*-value and fold change. GO and pathway analysis were performed based on the differentially expressed mRNAs.

### *In vivo* experimental evolution

Continuous passage of *P. aeruginosa* in a lung infection model was performed in neutropenic mice as previously described with minor modifications (21). Female C57BL/6N mice (6 to 8 weeks old) were intraperitoneally injected with 150 mg/kg and 100 mg/kg cyclophosphamide monohydrate (Aladdin, Shanghai, China) 72 hours and 24 hours before infection, respectively. Then the mice were anesthetized with isoflurane inhalation and intranasally inoculated with 5 × 10^8^ CFU of the clinical *P. aeruginosa* isolate CI-PA41. At 2 and 10 hours post infection (hpi), three mice were intraperitoneally injected with 100 mg/kg ceftazidime and 25 mg/kg avibactam (single drug group), while the other three mice were injected with ceftazidime-avibactam together with 300 mg/kg AZM (dual drug group). At 20 hpi, the mice were euthanized by CO_2_, and lungs of the mice were dissected and homogenized in 1 mL of 1% proteose peptone (G-CLONE, Beijing, China). The bacterial counts were determined by serial dilution and plating on LB agar. All of the remaining bacteria were spread on LB agar. After incubation at 37℃ for 16 hours, the bacteria were scraped off the LB plates and resuspended in LB medium. A portion of the bacteria was washed and resuspended in normal saline and used for the next round of *in vivo* passage. Another portion was serially diluted, and 15 µL suspension of each diluted culture was spotted on LB agar without or with CZA at the concentrations of 4, 8, and 16 mg/L. After incubation at 37 ℃ for 16 hours, colonies were counted to calculate CFU per milliliter. The fraction of bacteria resistant to a certain concentration (Cc; Cc >0) of CZA was calculated as previously described: (CFU/mL at Cc − CFU/mL at all concentrations above Cc)/total CFU (21). The rest of the bacteria were stored at −80℃.

## RESULTS

### Azithromycin represses resistance evolution to CZA in *P. aeruginosa*

**Fig 1:**
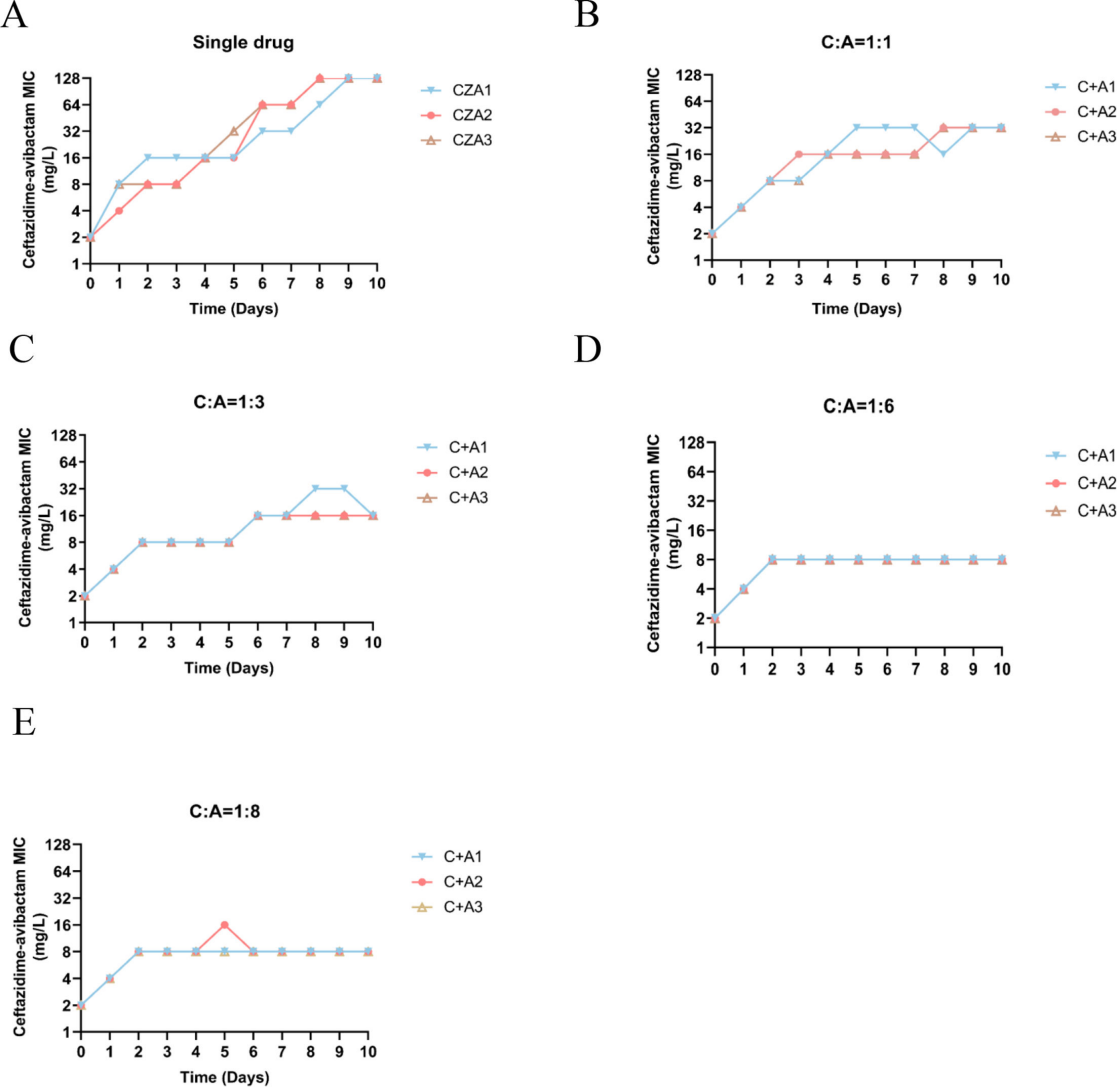
Development of CZA resistance by wild-type PA14. (**A**) Dynamics of stepwise development of resistance to CZA. Three parallel repeats were performed in the passaging, labeled CZA1–3. (**B–E**) Determination of the MICs of CZA over the passaging when combined with AZM at indicated ratios. C, CZA; A, azithromycin.

As in previous studies, the concentration of avibactam was fixed at 4 mg/L (18), and the concentrations of CZA hereafter represent those of ceftazidime. The antibiotics were combined at the ratios based on individual plasma concentrations (22–27). Combination of CZA with azithromycin in a 1:1 ratio displayed the most effective repression of resistance development (Fig. S1A and B; Fig. 1A and B).

**Fig 2:**
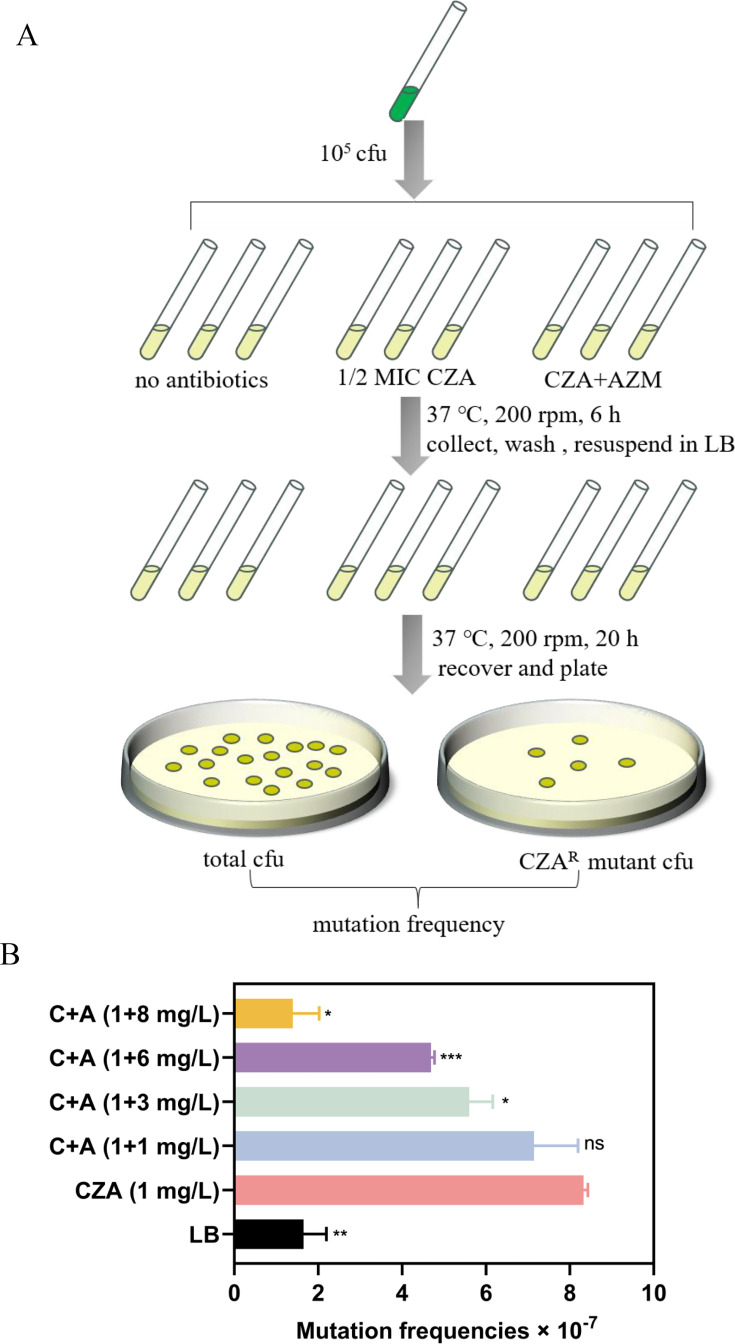
Mutation frequencies of resistance to CZA. (**A**) The experimental schematic diagram for measuring mutation frequencies. 10^5^ CFU of PA14 were incubated with indicated antibiotics for 6 hours. The bacteria were then resuspended in fresh LB medium without antibiotic and recovered for 20 hours, followed by serial dilution and plating on LB plates with or without CZA (16 mg/L). (**B**) The frequency of CZA resistance mutation. *, *P* < 0.05, **, *P* < 0.01, ***, *P* < 0.001, ns, not significant, compared to the cells treated with 1 mg/L CZA by Student’s *t*-test. C, CZA; A, azithromycin.

Azithromycin is enriched by phagocytes, and its concentration can reach up to 30 mg/L in infection sites, as evidenced by sputum specimens from cystic fibrosis (CF) patients (24). We thus increased the ratio of CZA to azithromycin from 1:1 up to 1:8. At the ratios of 1:6 and 1:8, the MIC of CZA remained at 8 mg/L after passaging for 10 days, whereas the MIC showed an increase when lower amounts of azithromycin were used (Fig. 1C through E). It has been demonstrated that subinhibitory concentrations of antibiotics provoke mutagenesis in bacteria (20, 28). We therefore examined the effect of CZA and azithromycin on bacterial mutation frequency using the method as previously described (20). Incubation of 10^5^ CFU of PA14 with 1 mg/L (1/2 MIC) CZA increased the frequency of resistance due to mutation (Fig. 2A and B), and the presence of azithromycin reduced the mutation frequency in a dose-dependent manner (Fig. 2B). In addition, the CZA-azithromycin combination repressed resistance development in 11 carbapenem-resistant *P. aeruginosa* clinical isolates, including CI-PA41 (Fig. S2).

### Azithromycin alters the expression of genes involved in stress-induced mutagenesis

We next explored the mechanism of the azithromycin-mediated repression of resistance development by transcriptome analysis. To obtain sufficient cells for RNA-Seq, 10^7^ CFU of wild-type PA14 were used in the assay. In order to identify the condition that elicits obvious bacterial response without killing the cells, we treated the bacteria with increasing concentrations of CZA and azithromycin, followed by determination of bacterial survival, mutation frequency, and the expression level of *ampC*, which responds to β-lactam antibiotics (29, 30). At the doses of up to 3 mg/L CZA and 16 mg/L (1/8 MIC) azithromycin, the live bacterial number remained unchanged after 12 hours (Fig. S3A through D). At the concentration of 3 mg/L, CZA induced the expression of *ampC* by sixfold compared to the control (Fig. S4A), and increased the resistance mutation frequency, which was reduced by 16 mg/L azithromycin (Fig. S4B and C).

**Fig 3:**
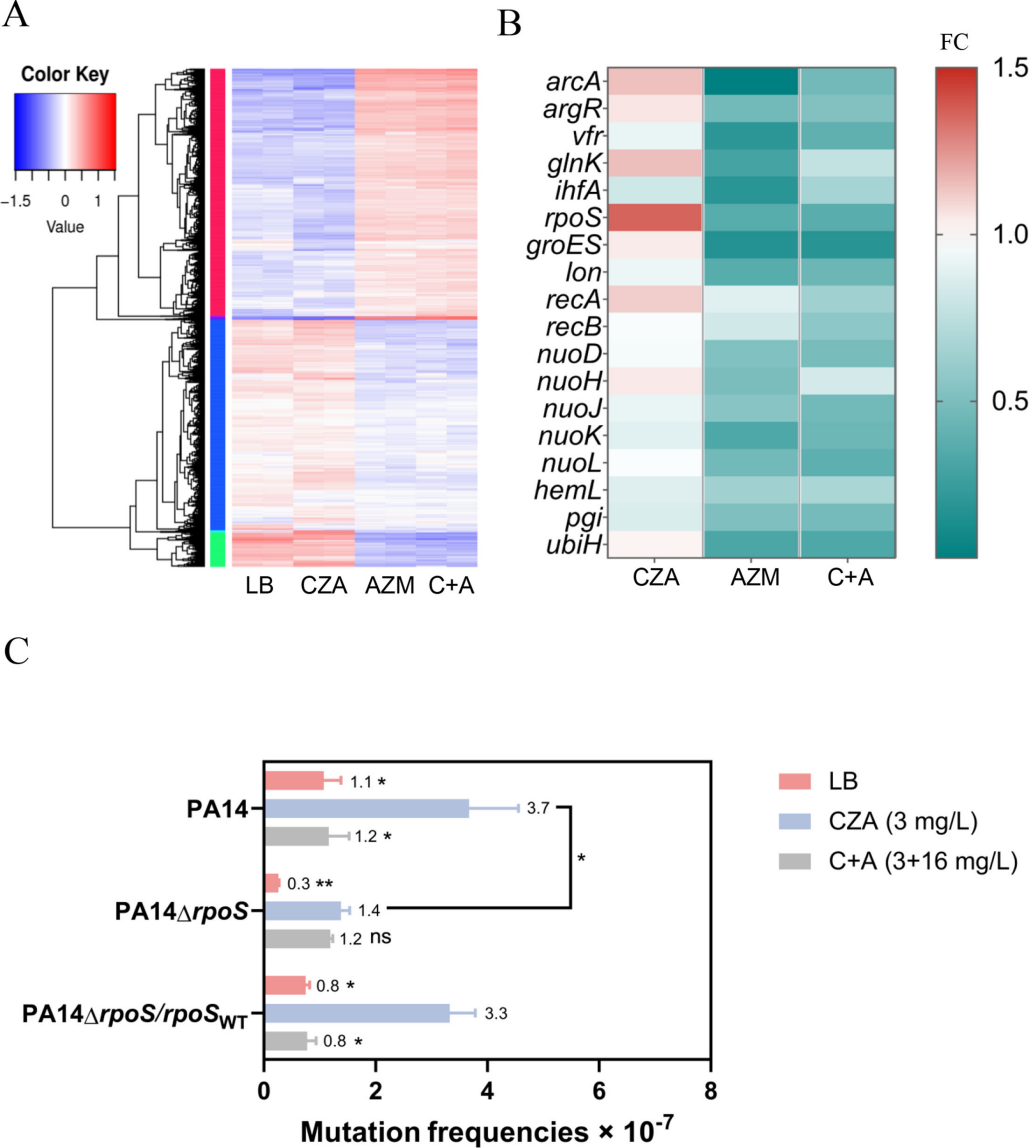
Azithromycin affects global gene expression. 10^7^ CFU of wild-type PA14 was grown in LB or LB containing 3 mg/L CZA, 16 mg/L AZM, or both of the drugs for 3 hours. Total RNA was extracted for RNA sequencing and analysis (**A**) or for qRT-PCR to determine the expression levels of genes related to stress-induced mutagenesis (**B**). C+A, CZA in combination with AZM. Role of RpoS in the mutation frequency of CZA resistance (**C**). Wild-type PA14, the Δ*rpoS* mutant, and the complemented strain (Δ*rpoS*/*rpoS*_WT_) were grown in LB or LB containing 3 mg/L CZA alone or in combination with 16 mg/L azithromycin (C+A). The frequency of CZA-resistant (CZA^R^) mutations was assessed. C+A, CZA in combination with AZM. *, *P* < 0.05, **, *P* < 0.01, ns, not significant, compared to the cells treated with 3 mg/L CZA by Student’s *t*-test. C, CZA; A, azithromycin.

Based on the above results, we treated 10^7^ CFU of wild-type PA14 with 3 mg/L CZA, 16 mg/L azithromycin alone or in combination for 3 hours before transcriptome analysis. Compared to the bacteria without antibiotic treatment, 153, 1,216, and 1,304 genes were upregulated, while 161, 1,277, and 1,324 genes were downregulated by CZA, azithromycin, and the combination, respectively (Fig. 3A; Table S5).

Previous studies with azithromycin identified genes related to stress-induced mutagenesis in *Escherichia coli* (31). Treatment of PA14 with azithromycin downregulated 18 mutation-promoting homologous genes (Table S6), which was verified by qRT-PCR (Fig. 3B). Among the azithromycin-repressed genes, the alternative sigma factor gene *rpoS* plays an important role in promoting antibiotic resistance in *E. coli* (20, 32). Deletion of *rpoS* in PA14 decreased both the background level and induced mutation frequency of resistance to CZA (Fig. 3C). In addition, mutation of *rpoS* reduced the expression levels of most mutation-promoting genes shown in *E. coli* (31), such as *arcA*, *argR*, *imuC*, *lon*, *nuoK*, *pgi*, *recB* (Fig. S5). These results indicate a role of RpoS in the resistance development.

### Azithromycin represses the translation of *rpoS* through 5´-terminal rare and less frequent codons

**Fig 4:**
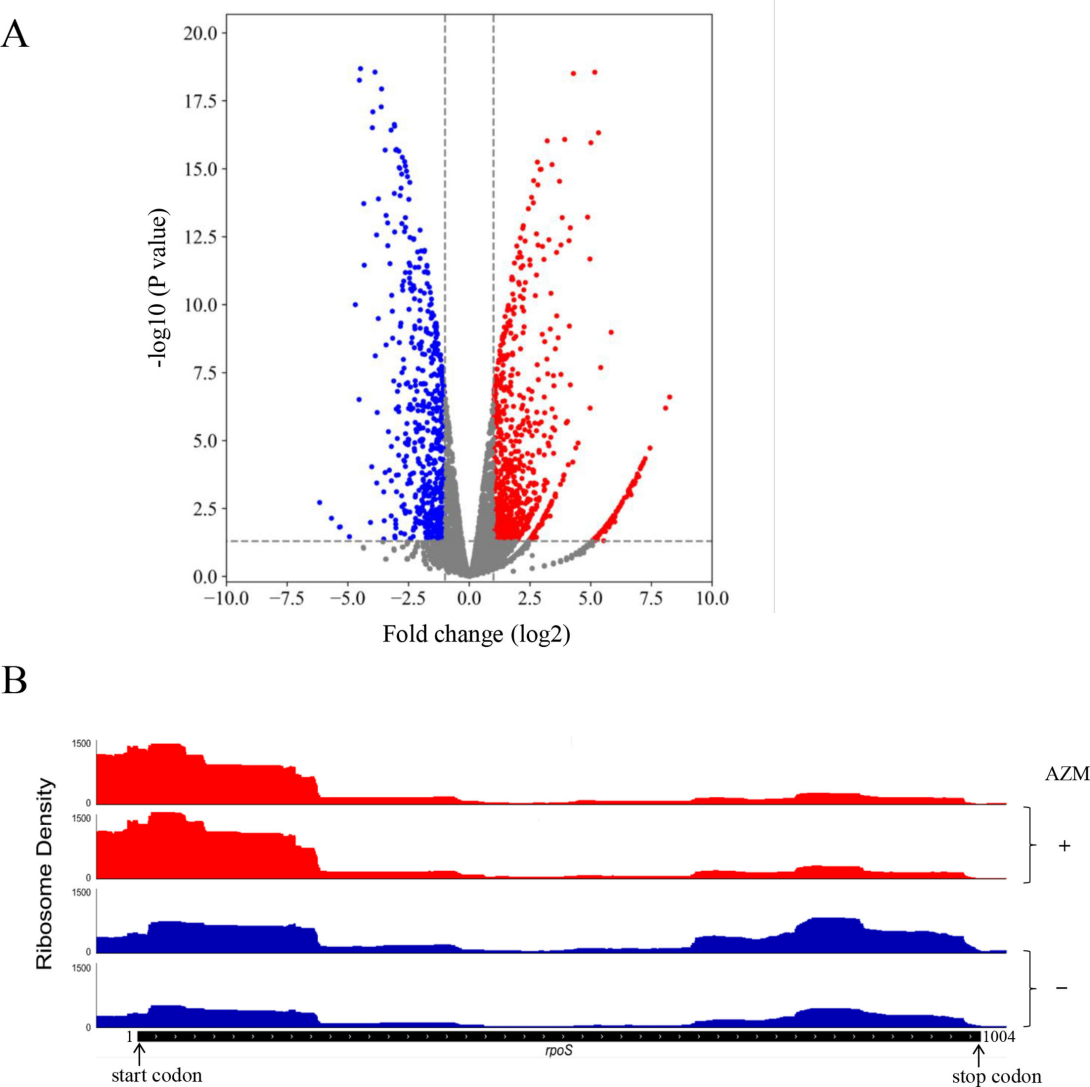
Redistribution of ribosome on cellular mRNA following azithromycin treatment. (**A**) Volcano plot depicting the mRNA counts protected by ribosome with or without azithromycin (16 mg/L) treatment. The *x*-axis of the graph shows log_2_-fold changes in mRNA counts (AZM-treated cells vs untreated cells). Red and blue dots represent significantly increased and decreased ribosome-associated mRNAs, respectively. (**B**) Ribosome distribution on the *rpoS* mRNA in wild-type PA14 grown in LB (blue) or LB containing azithromycin (red). The start and stop codons of *rpoS* were indicated by arrows.

Azithromycin binds to the nascent peptide exit tunnel (NPET) close to the peptidyltransferase center of the ribosome, which obstructs the NPET and subsequently induces ribosome stalling and depletion of intracellular pools of tRNAs (33, 34). To understand the mechanism through which azithromycin represses the transcription of mutation-associated genes, wild-type PA14 was treated with azithromycin, followed by determination of redistribution of ribosomes on the cellular mRNA by ribosome profiling. Azithromycin treatment increased ribosome stalling on 876 mRNAs, while it reduced stalling on 597 mRNAs (Fig. 4A).

Previous reports demonstrated that early elongation plays a critical role in the control of mRNA translation (35, 36). We thus calculated the ratio of the ribosome occupancy densities between the first 20 codons and the whole mRNA, which represents the extent of ribosome occupancy at the 5′-terminus (Table S7). Compared to cells grown in LB, azithromycin treatment resulted in higher (>1.5-fold) 5′-terminal ribosome occupancy in more than 500 genes, demonstrating a global inhibition of translation initiation.

**Fig 5:**
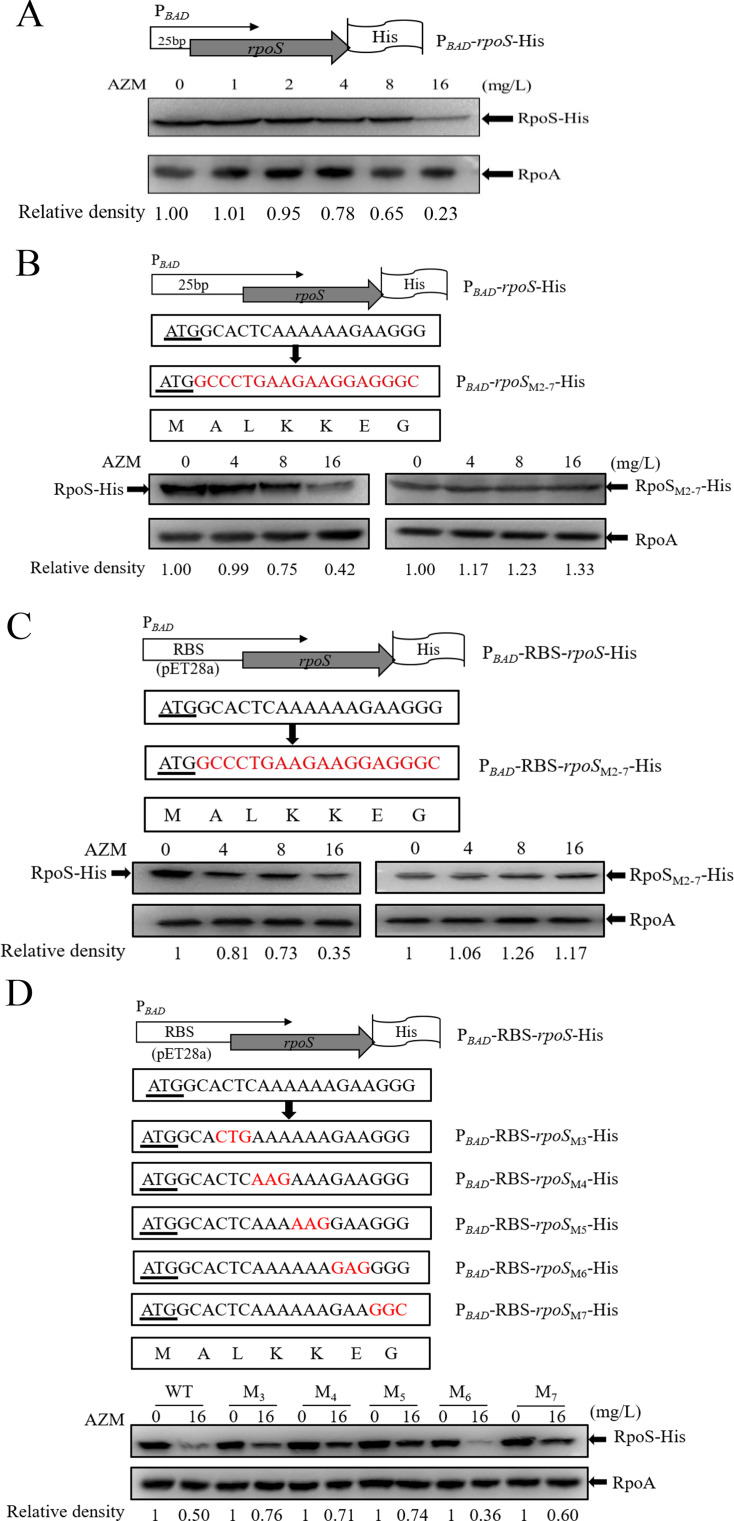
Azithromycin affects the translation of *rpoS*. (**A**) Structure of the 6×His-tagged *rpoS* (*rpoS*-His). Expression of C-terminal 6×His-tagged *rpoS* with its native 25-nucleotide sequence of the 5´ untranslated region is driven by an exogenous arabinose-inducible promoter (P*_BAD_*). PA14 containing the *rpoS*-His fusion was cultured in LB containing 0.1% L-arabinose and azithromycin at the indicated concentrations. When the OD_600_ reached 1.0, the bacteria were collected, and the RpoS-His levels were determined by western blot. (**B–D**) PA14 containing the indicated *rpoS*-His fusions was cultivated to an OD_600_ of 1 in the presence of 0.1% L-arabinose and azithromycin at indicated concentrations. Levels of the RpoS-His and RpoA (as loading control) were determined by western blot. (**B**) The first two to seven codons of *rpoS* in the P*_BAD_-rpoS*-His fusion were replaced with the corresponding common codons, resulting in P*_BAD_-rpoS*-M_2-7_-His. The usage frequencies of the first two to seven codons (shown in red) are 61.8%, 61.45%, 34.61%, 34.61%, 37.08%, and 70.81%, respectively, all representing the most frequently used codons. (**C**) The *rpoS*-His fusion with an exogenous ribosome-binding site from pET28a was driven by the P*_BAD_* promoter, resulting in P*_BAD_*-RBS-*rpoS*-His. Then the first two to seven codons of *rpoS* were replaced with the corresponding common codons (P*_BAD_*-RBS-*rpoS*-M_2-7_-His). (**D**) The individual codons at positions 2–7 of the P*_BAD_*-RBS-*rpoS*-His were replaced with the corresponding common codons (shown in red). The start codon (ATG) of *rpoS* is underlined.

Among the genes whose translation initiation is inhibited by azithromycin, we identified *rpoS*. Treatment with azithromycin increased the ribosome density at the N-terminus of the *rpoS* mRNA and reduced the ribosome density at the C-terminus (Fig. 4B). Since RpoS directly activates its own transcription (37), repression of translation by azithromycin might contribute to the reduction of the *rpoS* mRNA (Fig. 3B). To verify the effect of azithromycin on translation of *rpoS,* we utilized a C-terminal 6×His-tagged *rpoS* gene driven by an exogenous P*_BAD_* promoter (P*_BAD_-rpoS*-His, Fig. 5A). Azithromycin repressed the synthesis of RpoS-His in a dose-dependent manner (Fig. 5A).

Gödeke et al. demonstrated that AZM-induced ribosome stalling depletes the tRNA pool, intensifying repression of the translation of proteins with rare codons compared to those with frequent ones (33). By analyzing the initial codon sequence of *rpoS*, we found that the second, fourth, fifth, and seventh codons are classified as rare codons in *P. aeruginosa* (38), each representing less than 10% of the synonymous codon groups. The third and sixth codons are less frequently utilized but do not fall into the category of rare codons. Since azithromycin increased the ribosome occupancy at the N-terminus of the *rpoS* mRNA, we investigated whether those codons are involved in the translational repression. Replacement of the codons 2–7 with common codons (P*_BAD_-rpoS*_M2-7_-His) conferred resistance to the azithromycin-mediated translational repression (Fig. 5B).

Previous studies in *E. coli* revealed that the untranslated region (5′-UTR) of *rpoS* forms a hairpin structure with the coding region, which is involved in translational regulation (39). Alteration of the N-terminal codons of *rpoS* might influence the mRNA’s secondary structure. To examine whether the 5′-UTR is involved in the azithromycin-mediated translational repression, we replaced the native 5′-UTR with the ribosome-binding site-containing sequence from the commercial vector pET28a (P*_BAD_*-RBS-*rpoS*-His, Fig. 5C). The translation of the resultant *rpoS*-His fusion with native codons was repressed by azithromycin, whereas the one with the common codons displays resistance to the repressions (Fig. 5C). For an unknown reason, the codon 2–7 mutation reduced the protein level in the absence of azithromycin (Fig. 5B and C). Nevertheless, these results demonstrate that azithromycin mainly represses the translation of *rpoS* through the first 2–7 codons.

To dissect the contribution of each of the codons in the azithromycin-mediated translational repression, we replaced the individual codons with the corresponding common ones in the *rpoS*-His fusion with the exogenous P*_BAD_* promoter and 5′-UTR (Fig. 5D). Replacement of the second codon diminished the translation of the *rpoS* mRNA in an unclear way (Fig. S6). Replacement of the third, fourth, and fifth codons increased the protein synthesis by around 1.5-fold compared to the wild-type sequence in the presence of azithromycin, demonstrating increased resistance to the azithromycin-mediated translational repression (Fig. 5D).

**Fig 6:**
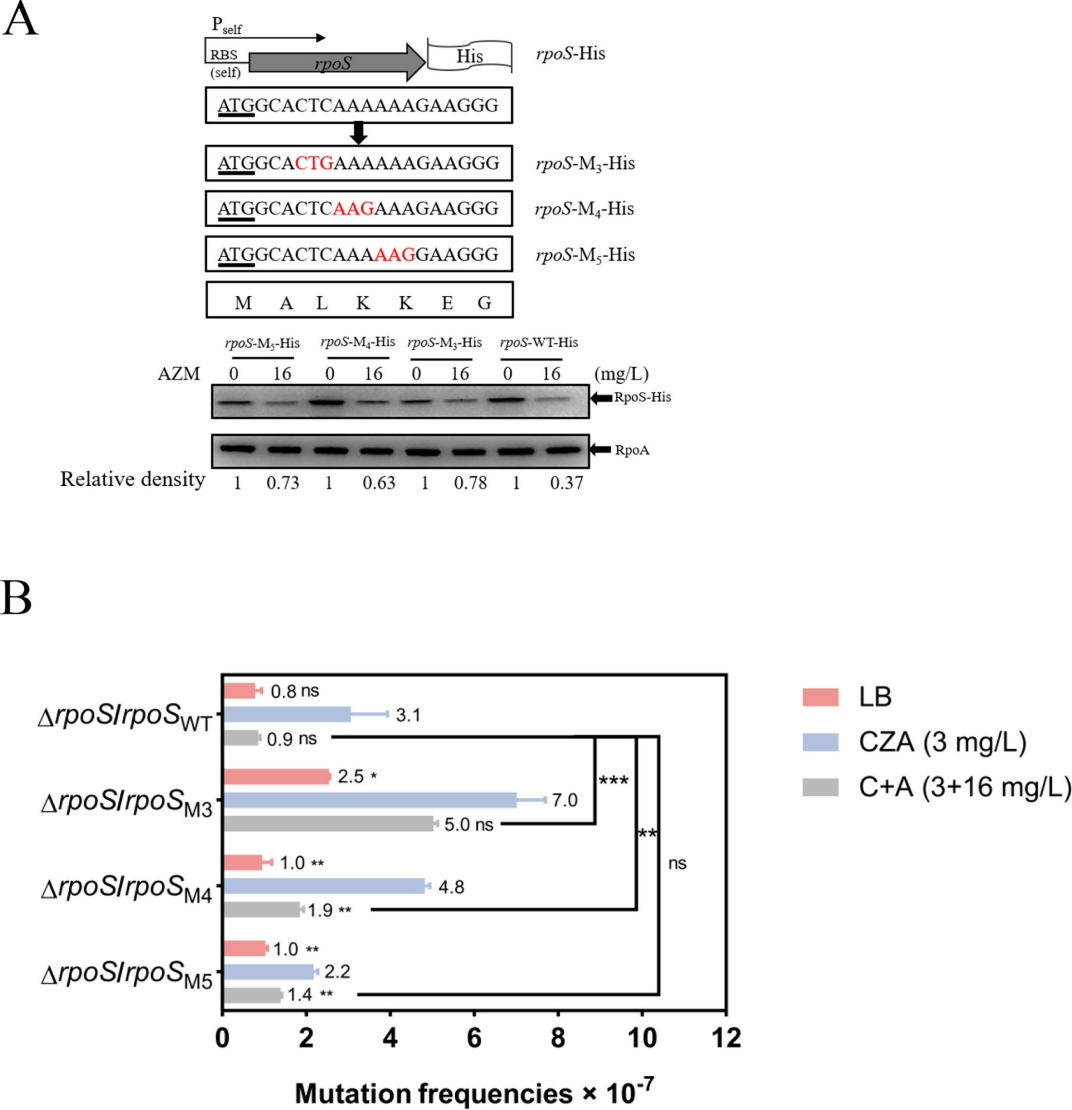
Roles of the N-terminal codons of *rpoS* in the azithromycin-mediated repression of translation and mutation frequency. (**A**) In the *rpoS*-His with the native 5´-UTR and promoter, the third, fourth, and fifth codons were individually changed to the corresponding common ones, resulting in *rpoS*-M_3_-His, *rpoS*-M_4_-His, and *rpoS*-M_5_-His. PA14 containing the indicated *rpoS*-His fusions was cultivated to an OD_600_ of 1 in the presence of 0.1% L-arabinose and azithromycin at indicated concentrations. Levels of the RpoS-His and RpoA were determined by western blot. (**B**) The Δ*rpoS* mutant complemented with wild-type *rpoS* (*rpoS*-His) or the mutated *rpoS* (*rpoS*-M_3_-His, *rpoS*-M_4_-His, and *rpoS*-M_5_-His) was grown in LB or LB containing 3 mg/L CZA alone or in combination with 16 mg/L azithromycin (C+A). The frequency of CZA-resistant (CZA^R^) mutations was assessed. *, *P* < 0.05, **, *P* < 0.01, ns, not significant, compared to the cells treated with 1 mg/L CZA by Student’s *t*-test. The start codon (ATG) of *rpoS* is underlined. C, CZA; A, azithromycin.

We then examined the effects of the point mutations on the bacterial mutation frequency in the presence of azithromycin. First, we replaced the wild-type low-frequency third, fourth, and fifth codons with more common ones in the *rpoS*-His with its endogenous promoter and 5′-UTR, resulting in *rpoS*-M3-His, *rpoS*-M4-His, and *rpoS*-M5-His (Fig. 6A). Compared to the wild-type *rpoS*, the point mutations resulted in 1.8- to 2.1-fold increased resistance to the azithromycin-mediated repression of translation (Fig. 6A). We then complemented the Δ*rpoS* strain with *rpoS*-His or with the individual point mutants in *rpoS*-His. As for the Δ*rpoS* mutant complemented with the *rpoS*-His, CZA increased the resistance mutation frequency by 3.88-fold (from 0.8 × 10^−7^ to 3.1 × 10^−7^), which was reduced to the background level by azithromycin (Fig. 6B). Complementation with the *rpoS*-M3-His and *rpoS*-M4-His increased the CZA-induced mutation frequencies to 7.0 × 10^−7^ and 4.8 × 10^−7^, and in the presence of azithromycin, the mutation frequencies were 5.0 × 10^−7^ and 1.9 × 10^−7^, which were 5.56- and 2.11-fold higher than that of the Δ*rpoS*/*rpoS*-His, respectively (Fig. 6B). Collectively, these results demonstrate that switching the third and fourth codons to common ones increased resistance to the azithromycin-mediated suppression of resistance mutation.

### Repression of resistance development by the CZA-azithromycin combination *in vivo*

**Fig 7:**
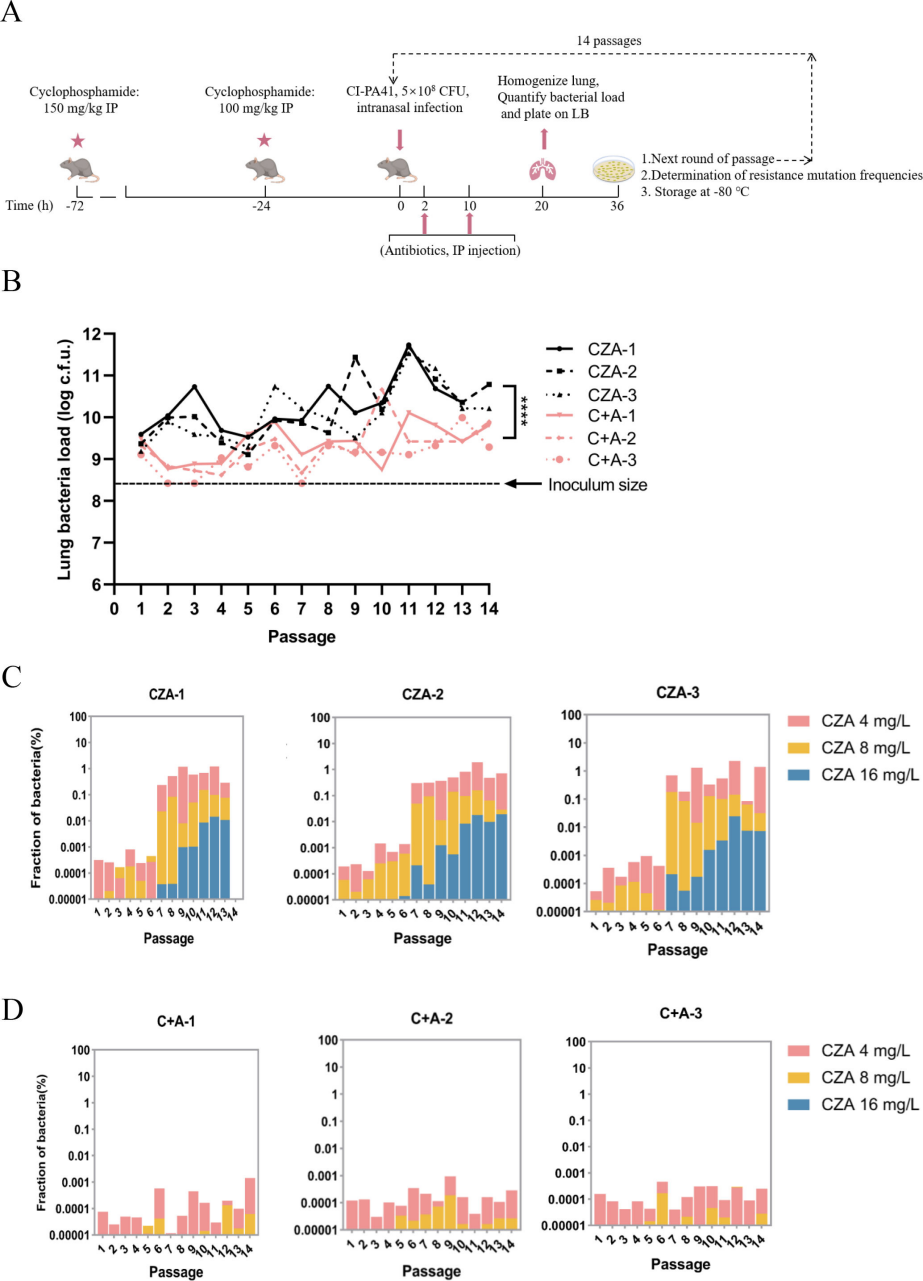
Development of CZA resistance *in vivo*. (**A**) The *in vivo* passage in the neutropenic mice using the pneumonia model. At 72 and 24 hours before infection, the mice were injected with cyclophosphamide. At the time of infection, the mice were intranasally inoculated with 5 × 10^8^ CFU of the clinical *P. aeruginosa* isolate CI-PA41. At 2 and 10 hours post-infection, the mice were intraperitoneally injected with CZA alone or in combination with azithromycin. At 20 hours post-infection, the mice were euthanized, and bacteria in the lungs were collected, followed by serial dilution and plating on LB agar plates for CFU counting (**B**). All of the remaining bacteria were plated on LB plates. After 16 hours, bacteria were scraped from the plates and resuspended for the next round of passage (**A**). (**C, D**) Another portion of the bacterial suspension was serially diluted, and 15 µL of the bacteria suspension was spotted on LB plates with or without CZA to determine the resistance mutation frequencies. The limit of detection was 67 CFU/mL. ****, *P* < 0.0001, analysis was conducted using the one-way analysis of variance test. C, CZA; A, azithromycin.

Recently, Huo et al. demonstrated that *Acinetobacter baumannii* quickly developed resistance in neutropenic mice receiving ciprofloxacin treatment in a pneumonia model (21). We adopted the method to examine the effect of the CZA-azithromycin combination on the resistance development *in vivo* (Fig. 7A). Since CZA is mainly used to treat carbapenem-resistant bacteria-caused infections, we utilized the previously reported clinical isolate CI-PA41 (Table S8). The strain was passaged by intranasal inoculation in neutropenic mice. At 2 and 10 hours post-infection, the mice were injected with CZA alone or together with azithromycin. At 20 hours post-infection, bacteria were isolated from the lungs of the euthanized mice and plated on LB plates, which were used as inocula for the next round of infection. In the mice that received CZA treatment, the bacterial loads displayed a trend of increasing over the course of passages (Fig. 7B). In the 14th passage, one of the three CZA-treated mice died, and the other two mice showed severe signs of disease. We thus stopped the passaging experiment. In contrast, the bacterial loads in the azithromycin-CZA treated mice remained relatively stable (Fig. 7B).

To investigate the impact of azithromycin on the development of resistance to CZA *in vivo*, we quantified the resistance frequency by plating the isolated bacteria on plates without or with increasing concentrations of CZA (Fig. 7C and D). The results revealed a progressive increase in the fraction of CZA-resistant bacteria in the CZA monotherapy group over the passages, while the CZA-azithromycin dual-drug treatment group maintained a low proportion of resistance (Fig. 7C and D). From passage 7, clinically resistant mutants (MIC of CZA ≥16 mg/L) appeared in the monotherapy groups, and the proportions kept increasing afterward (Fig. 7C and D). In the dual-drug treatment groups, the CZA resistance mutation frequencies of MIC ≥4 mg/L and ≥8 mg/L were 1,614- and 781-fold lower than those of the monotherapy groups (passage 14), respectively, and no colony appeared on the plates containing 16 mg/L CZA (Fig. 7C and D). Collectively, these results demonstrate a suppressive effect of azithromycin on the development of CZA resistance *in vivo*.

## DISCUSSION

Currently, bacterial resistance evolution outpaces drug development (40). This highlights the critical need for strategies that can slow down the development of antibiotic resistance, thereby extending the useful lifespan of existing and novel antibiotics.

In this study, we demonstrated that the combination of azithromycin and CZA effectively inhibits the evolution of resistance to CZA in *in vitro* and *in vivo*.

The sigma factor RpoS is a conserved key regulator of the general stress response in proteobacteria (41). RpoS plays an important role in promoting antibiotic resistance and is involved in stress-induced mutagenesis in *E. coli* (20). Here, we found that deletion of *rpoS* in wild-type *P. aeruginosa* PA14 decreased both the basal and induced mutation frequencies of CZA resistance.

Previously, Kannan et al. utilized erythromycin and telithromycin at the doses of 100-fold of the MICs to treat *E. coli* before the ribosome profiling assay and found the most prevalent ribosome stalling motif was found to be [R/K]X[R/K] (42). Later, Davis et al. treated *Staphylococcus aureus* with azithromycin at the concentration 200-fold higher than the MIC for 15 minutes before performing ribosome profiling assay and found the major stalling sequence is (R/K)x(R/K) and xPx (43). While high concentrations of azithromycin can quickly arrest translation, subinhibitory concentrations result in depletion of the intracellular pools of tRNAs in *P. aeruginosa*, which might affect the translation of rare codon-containing genes (33). Consistent with this concept, azithromycin at 2 mg/L repressed the translation of *rhlR*, and replacement of the rare codon of the second amino acid conferred resistance to the translational repression (33, 44).

In our ribosome profiling assay, azithromycin was used at the same concentration as in the assays for mutation frequency and gene expression (16 mg/L, 1/8 MIC), which is also achievable *in vivo* in the clinic settings (24). We found repression of the initial elongation of *rpoS*. Replacement of the fourth rare codon and the third less frequent codon of *rpoS* with the corresponding frequent codons conferred resistance to the azithromycin-caused repression on translation and mutation frequency. Besides *rpoS*, azithromycin represses the translation initiation of around 500 genes, including other genes involved in gene regulation, metabolism, or transport. We are currently making efforts to analyze our ribosome profiling to identify genes whose translation is arrested in the internal regions or along the whole length.

Azithromycin has been shown to reduce the expression of virulence factors and QS regulators (15, 16). Additionally, RpoS, a key regulator of the stress response, is known to modulate the expression of virulence factors and other stress-related genes. In our study, azithromycin treatment resulted in a significant downregulation of *rpoS* expression. We propose that azithromycin may exert its effects by decreasing *rpoS* levels, thereby suppressing the expression of virulence and QS-related genes. However, additional experiments are required to validate this hypothesis.

Mutations in the *rpoS* gene are commonly found in clinical isolates of *P. aeruginosa*. These mutations have been shown to result in altered stress responses, enhanced biofilm formation, and increased resistance to various antibiotics. Previous studies highlight the accumulation of point mutations in the *rpoS* gene in clinical isolates, which may have significant implications for treatment outcomes (45). In this study, we observed that the combination of azithromycin and CZA had varying levels of efficacy across different strains of *P. aeruginosa*, potentially reflecting the presence of *rpoS* mutations. The presence of *rpoS* mutations may reduce the effectiveness of treatments that rely on disrupting stress responses or biofilm formation, suggesting that the presence of these mutations should be considered when selecting treatment regimens for clinical infections.

Development of CZA-resistant *P. aeruginosa* has been reported in a patient with neutropenia (10). By using a neutropenic mouse pneumonia model, we found that CZA-resistant *P. aeruginosa* mutants (MIC ≥16 mg/L) appeared on days 6 and 7 in the CZA-treated groups. However, in the groups treated with CZA-azithromycin combination, no resistant mutant was identified, and the bacterial loads remained stable during the passaging. Therefore, the combination of CZA with azithromycin might be a promising therapeutic strategy against *P. aeruginosa* infections.

## Data Availability

The transcriptome (RNA sequencing) data that support the findings of this study have been deposited in the NCBI Sequence Read Archive (SRA) with the accession code PRJNA1029762, the ribosome profiling data discussed in this publication have been deposited in NCBI’s Gene Expression Omnibus and are accessible through GEO Series accession number GSE245932 (https://www.ncbi.nlm.nih.gov/geo/query/acc.cgi?acc=GSE245932).

## SUPPLEMENTAL MATERIAL

Supplemental figures jb.00552-24-s0001.pdf Figure S1 to S6.

Supplemental tables jb.00552-24-s0002.docx Tables S1 to S4 and S6.

Table S5 jb.00552-24-s0003.xlsx Ratios of ribosome counts between the first 20 codons and the entire mRNA with or without azithromycin treatment.

Table S7 jb.00552-24-s0004.xlsx Global gene expression following the treatment with CZA, azithromycin, and CZA-azithromycin combination.

ASM does not own the copyrights to Supplemental Material that may be linked to, or accessed through, an article. The authors have granted ASM a non-exclusive, world-wide license to publish the Supplemental Material files. Please contact the corresponding author directly for reuse.
