# Supplementary material for: Azithromycin represses evolution of ceftazidime/avibactam resistance by translational repression of rpoS in Pseudomonas aeruginosa
Source: J Bacteriol. 2025 Apr 30;207(5):e00552-24. doi: 10.1128/jb.00552-24 (PMC12096824; doi:10.1128/jb.00552-24)
Supplement: Supplemental figures — Figure S1 to S6. [file jb.00552-24-s0001.pdf]

Figure S1

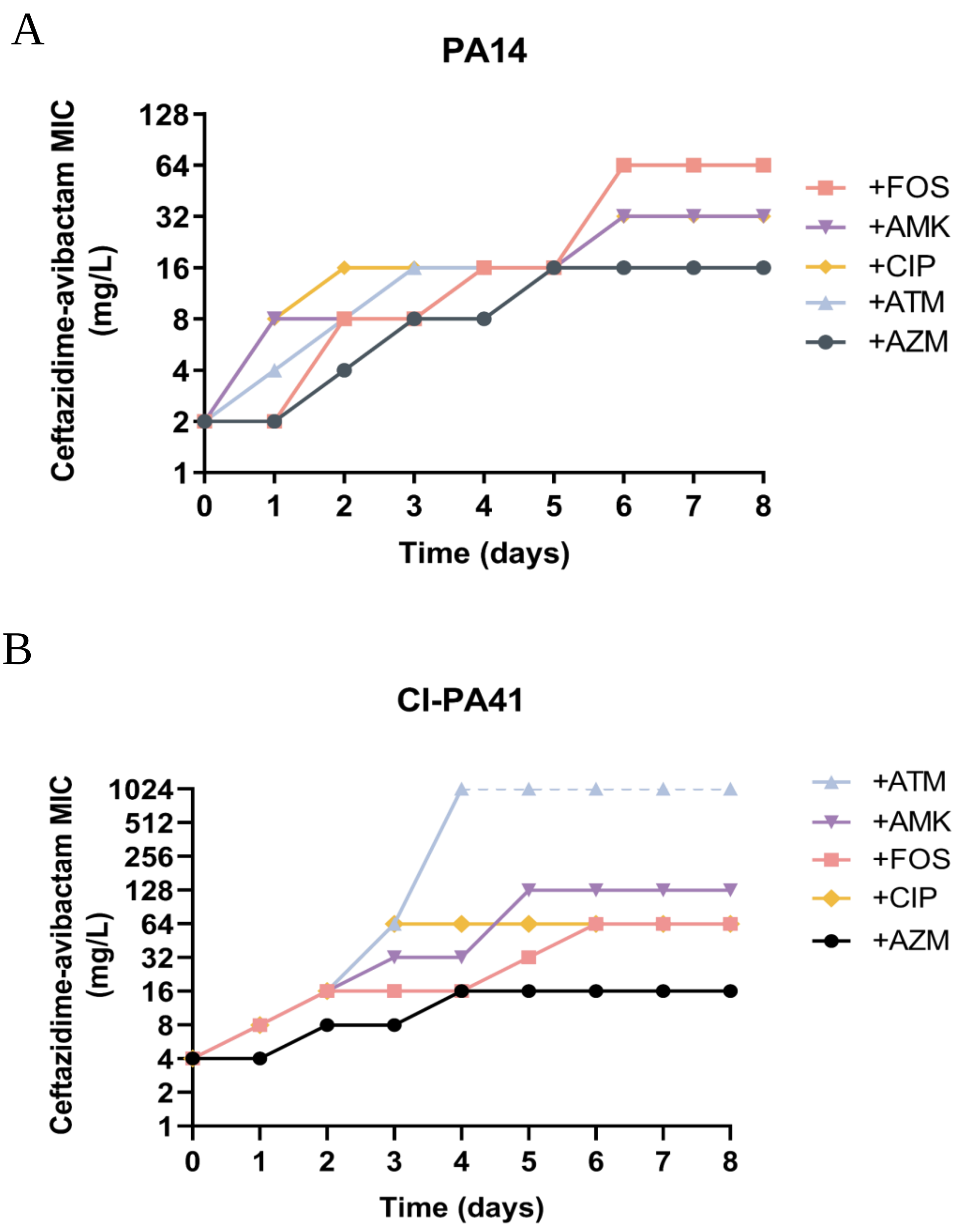

Figure S1. Development of CZA resistance in the *in vitro* passaging assay. (A) Resistance development of PA14 (A) and CI-PA41 (B) in the presence CZA alone or in combination with aztreonam (ATM), amikacin (AMK), azithromycin (AZM), fosfomycin (FOS), and ciprofloxacin (CIP). The combination ratios were as follows: CZA:ATM = 1:1, CZA:AMK = 2:1, CZA:AZM = 1:1, CZA:FOS = 1:1, and CZA:CIP = 10:1.

Figure S2

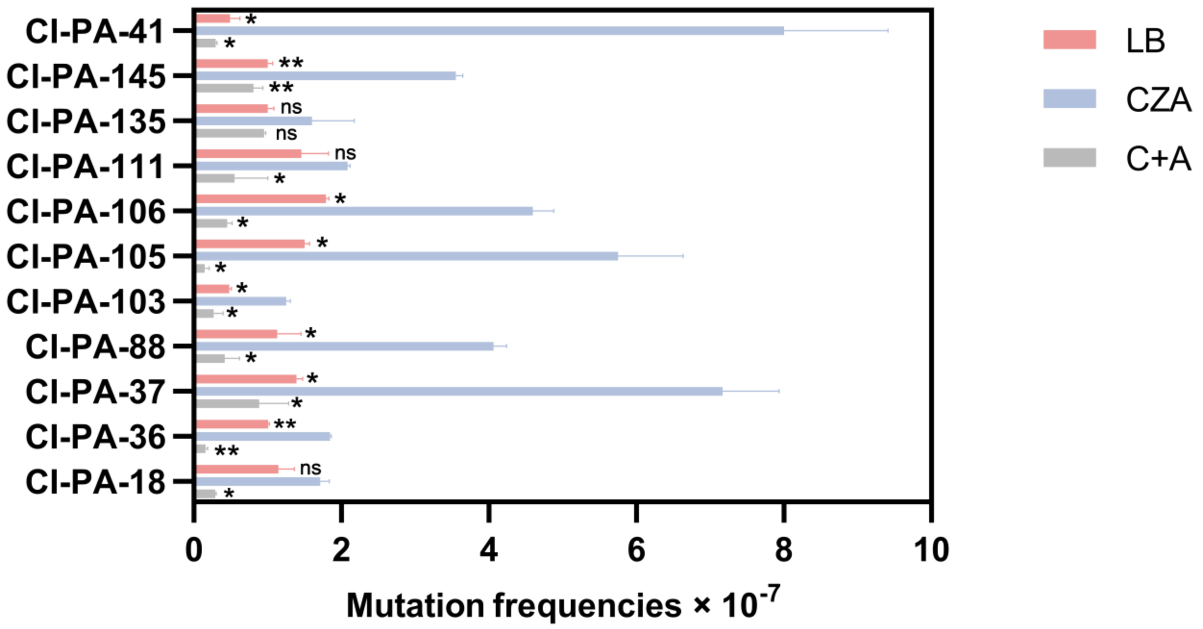

Figure S2. Repression of mutagenesis by the CZA-azithromycin combination in carbapenem-resistant *P. aeruginosa* clinical isolates. 10^5 CFU of the strains were incubated with CZA alone or in combination with azithromycin for 6 hours. Then the bacteria were resuspended in fresh medium and recovered for 20 hours, followed by serial dilution and plating on LB plates with or without CZA. For each strain, the ceftazidime-avibactam concentration was set at half of the individual MIC for the clinical isolates, with a ceftazidime-avibactam: AZM ratio of 1:6. C+A, CZA in combination with AZM.). *, P < 0.05, **, P < 0.01, ns, not significant, compared to the cells treated with 1 mg/L CZA by Student's t test.

Figure S3

A

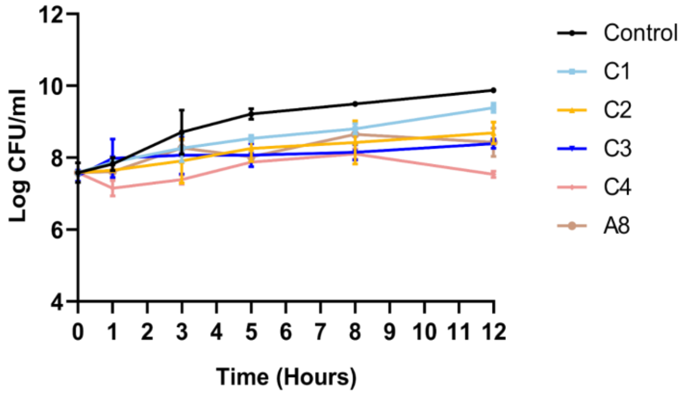

B

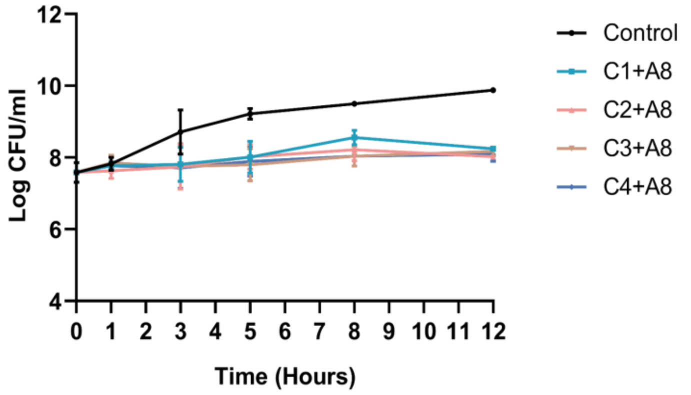

C

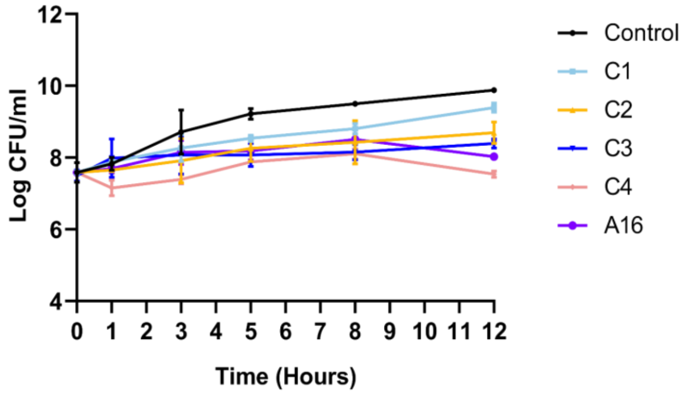

D

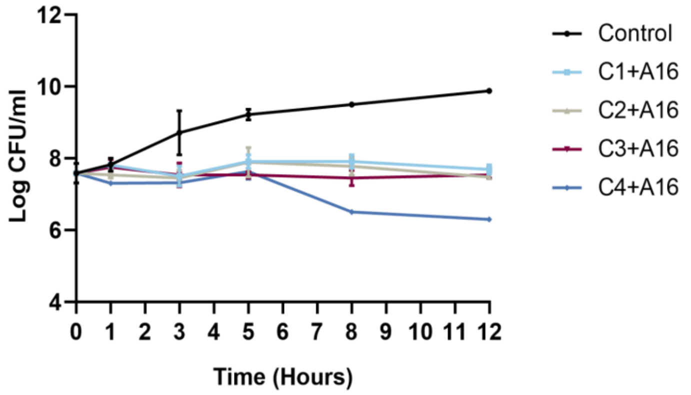

Figure S3. The overnight culture was transferred into 30 mL LB at a specific ratio to achieve a final concentration of 10^7 CFU/mL and subsequently exposed to CZA at the concentrations of 1, 2, 3, and 4 mg/L, AZM at the concentrations of 8 and 16 mg/L (A, C) or combination of the drugs at indicated concentrations (B, D). The bacterial survival was determined by serial dilution and plating on LB agar plates. Data represent the mean bacterial count (Log CFU/ml) and the error bars indicate the SD of the mean. All treatments were performed in triplicate. C, CZA; A, azithromycin. The number following the letter indicates the concentration (mg/L).

Figure S4

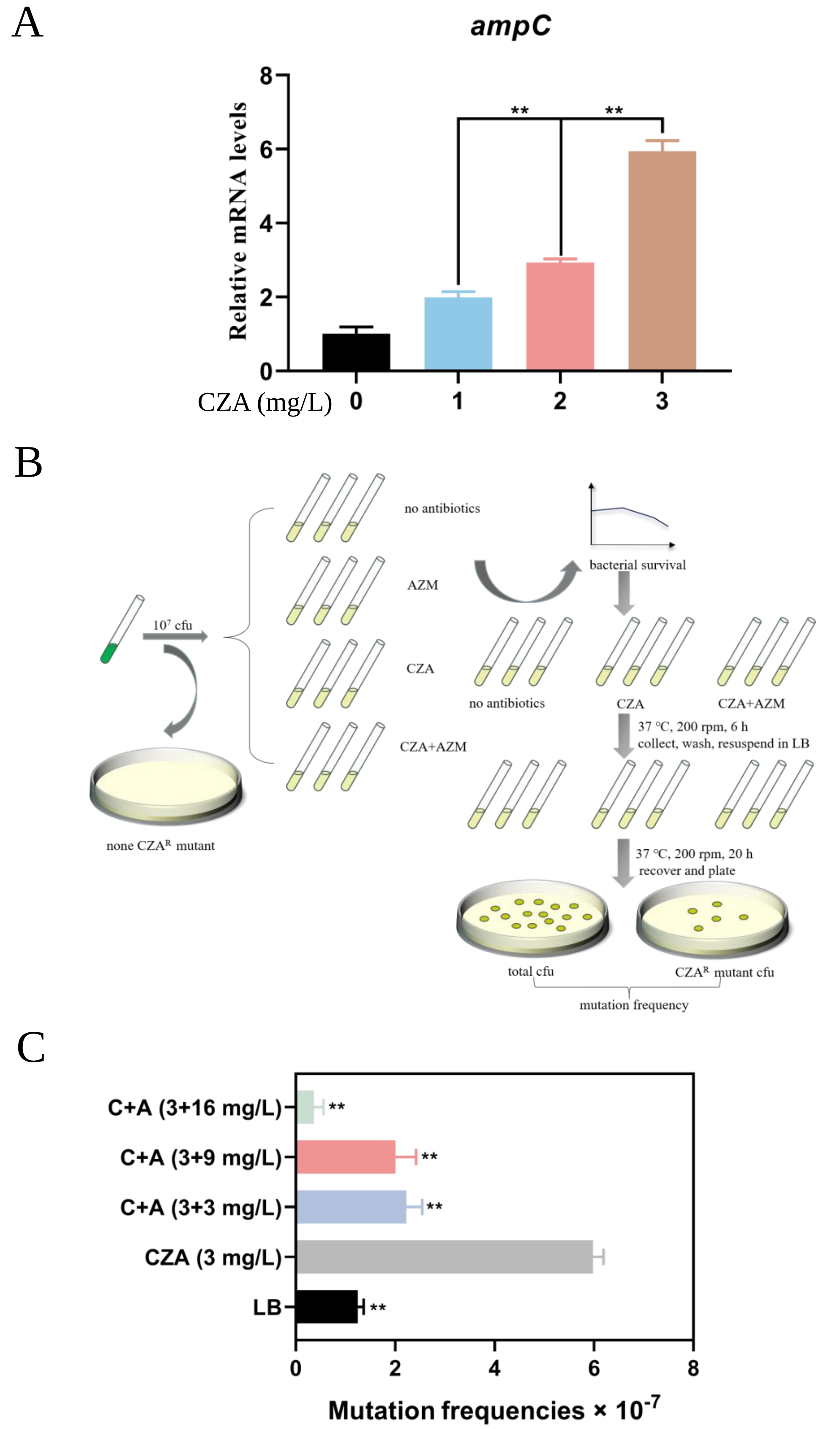

Figure S4. (A) The wild type PA14 was cultured in LB in the absence or presence of 1, 2, and 3 mg/L CZA to an OD_{600} of 1. The *ampC* mRNA levels were determined by qRT-PCR analysis. (B) The experimental schematic diagram for measuring mutation frequencies. 10^7 CFU of PA14 were incubated with indicated antibiotics for 6 hours. Then the bacteria were resuspended in fresh medium and recovered for 20 hours, followed by serial dilution and plating on LB plates with or without CZA. (C) The frequencies of CZA resistant (CZAR) mutations. C+A, CZA in combination with AZM. **, P < 0.01, by Student’s t test.

Figure S5

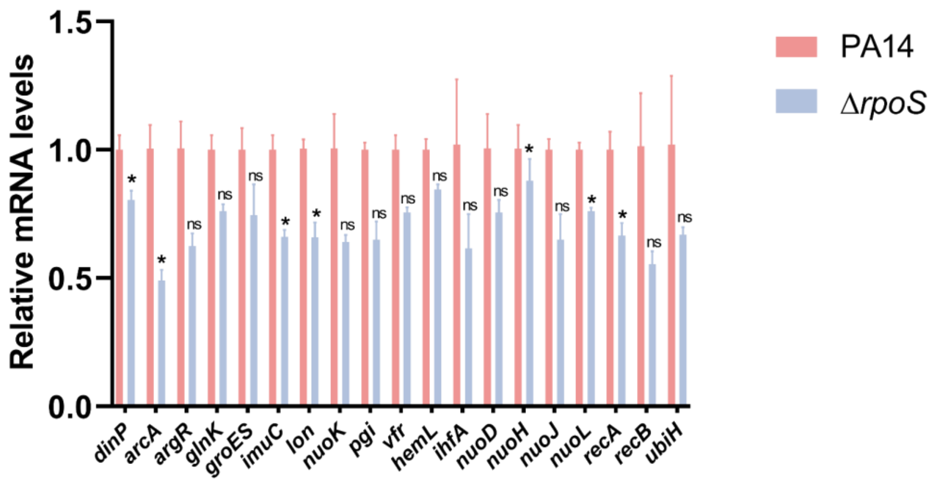

Figure S5. PA14 and Δ*rpoS* strain were grown to an OD_{600} of 1, total RNA was extracted for qRT-PCR to determine the expression levels of mutation-promoting genes.

Figure S6

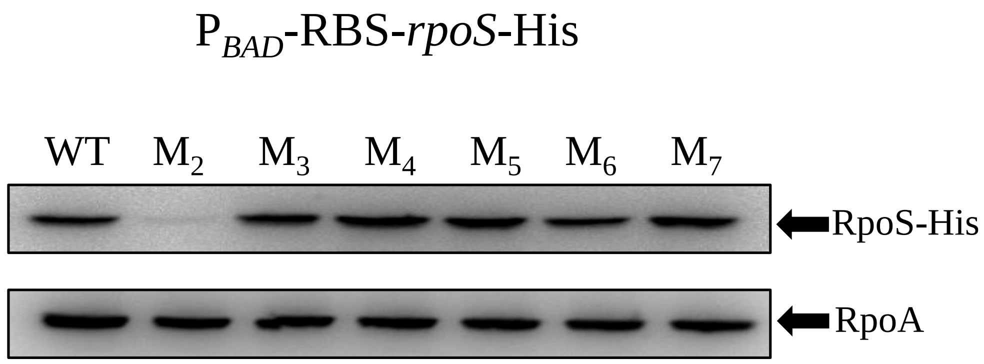

Figure S6. The individual codons at positions 2-7 of the P_{BAD}-RBS-*rpoS*-His was replaced with the corresponding common codons, resulting in M2 to M7. PA14 containing the indicated *rpoS*-His fusions was cultivated to an OD_{600} of 1 in the presence of 0.1% L-arabinose and the levels of the RpoS-His and RpoA were determined by western blot.
