# Supplementary material for: Azithromycin represses evolution of ceftazidime/avibactam resistance by translational repression of rpoS in Pseudomonas aeruginosa
Source: J Bacteriol. 2025 Apr 30;207(5):e00552-24. doi: 10.1128/jb.00552-24 (PMC12096824; doi:10.1128/jb.00552-24)
Supplement: Supplemental tables — Tables S1 to S4 and S6. [file jb.00552-24-s0002.docx]

**Table S1 Bacteria, plasmids, primers used in this study.**

|  | Description | | Source | |
| --- | --- | --- | --- | --- |
| Bacteria |  | |  | |
| PA14 | Wild type strain of *P. aeruginosa* | | Lab stock | |
| CI-PA-41 | *P. aeruginosa* clinical isolate | | Lab stock | |
| CI-PA-18 | *P. aeruginosa* clinical isolate | | This study | |
| CI-PA-36 | *P. aeruginosa* clinical isolate | | This study | |
| CI-PA-37 | *P. aeruginosa* clinical isolate | | This study | |
| CI-PA-88 | *P. aeruginosa* clinical isolate | | This study | |
| CI-PA-103 | *P. aeruginosa* clinical isolate | | This study | |
| CI-PA-105 | *P. aeruginosa* clinical isolate | | This study | |
| CI-PA-106 | *P. aeruginosa* clinical isolate | | This study | |
| CI-PA-111 | *P. aeruginosa* clinical isolate | | This study | |
| CI-PA-135 | *P. aeruginosa* clinical isolate | | This study | |
| CI-PA-145 | *P. aeruginosa* clinical isolate | | This study | |
| PA14Δ*rpoS* | PA14 deletion of *rpoS* | | This study | |
| PA14*rpoS*  */rpoS*_WT_-His | Δ*rpoS* with wild type *rpoS* fused His tag inserted on chromosome with mini-Tn7T insertion | | This study | |
| PA14Δ*rpoS*  */rpoS*_M3_-His | Δ*rpoS* with *rpoS* +3 codon mutant fused His tag inserted on chromosome with mini-Tn7T insertion | | This study | |
| PA14Δ*rpoS*  */rpoS*_M4_-His | Δ*rpoS* with *rpoS* +4 codon mutant fused His tag inserted on chromosome with mini-Tn7T insertion | | This study | |
| PA14Δ*rpoS*  */rpoS*_M5_-His | Δ*rpoS* with *rpoS* +5 codon mutant fused His tag inserted on chromosome with mini-Tn7T insertion | | This study | |
| PA14 RpoS-His | PA14 with His tag inserted at *rpoS* coding region before stop codon on chromosome | | This study | |
| Plasmids |  | |  | |
| pEX18Tc | Gene replacement vector; TET^r^, *oriT*^+^, *sacB*^+^ | | [1] | |
| pEX18Tc-Δ*rpoS* | *rpoS* gene deletion on pEX18Tc | | This study | |
| pUC18T-mini-Tn7T-Gm | Mini-Tn7 base vector from insertion into chromosome attTn7 site; GEN^r^ | | [1] | |
| pTNS3 | Helper plasmid, for gene insertion in chromosome; AMP^r^ | | [1] | |
| pUC18T-mini-Tn7T-Gm-*rpoS*_WT_ | pUC18T-mini-Tn7T-Gm with *rpoS*_WT_, GM^r^ | | This study | |
| pUC18T-mini-Tn7T-Gm-*rpoS*_M3_ | pUC18T-mini-Tn7T-Gm with *rpoS*_M3_, GM^r^ | | This study | |
| pUC18T-mini-Tn7T-GM-*rpoS*_M4_ | pUC18T-mini-Tn7T-GM with *rpoS*_M4_, GM^r^ | | This study | |
| pUC18T-mini-Tn7T-GM-*rpoS*_M5_ | pUC18T-mini-Tn7T-GM with *rpoS*_M5_, GM^r^ | | This study | |
| pRKaraRed | Expression vector with P_BAD_ promoter; TET^r^ | | [2] | |
| pRKaraRed-*rpoS*(F1)-His | *rpoS*(F1) fused to His on pRKaraRed; TET^r^ | | This study | |
| pRKaraRed-*rpoS*(F1)-M_2-7_-His | *rpoS*(F1)-M_2-7_ fused to His on pRKaraRed; TET^r^ | | This study | |
| pRKaraRed-*rpoS*(RBS)-His | *rpoS*(RBS) fused to His on pRKaraRed; TET^r^ | | This study | |
| pRKaraRed-*rpoS*(RBS)-M_2-7_-His | *rpoS*(RBS)-M_2-7_ fused to His on pRKaraRed; TET^r^ | | This study | |
| pRKaraRed-*rpoS*(RBS)-M_345_-His | *rpoS*(RBS)-M_345_ fused to His on pRKaraRed; TET^r^ | | This study | |
| pRKaraRed-*rpoS*(RBS)-M_2_-His | *rpoS*(RBS)-M_2_ fused to His on pRKaraRed; TET^r^ | | This study | |
| pRKaraRed-*rpoS*(RBS)-M_3_-His | *rpoS*(RBS)-M_3_ fused to His on pRKaraRed; TET^r^ | | This study | |
| pRKaraRed-*rpoS*(RBS)-M_4_-His | *rpoS*(RBS)-M_4_ fused to His on pRKaraRed; TET^r^ | | This study | |
| pRKaraRed-*rpoS*(RBS)-M_5_-His | *rpoS*(RBS)-M_5_ fused to His on pRKaraRed; TET^r^ | | This study | |
| pRKaraRed-*rpoS*(RBS)-M_6_-His | *rpoS*(RBS)-M_6_ fused to His on pRKaraRed; TET^r^ | | This study | |
| pRKaraRed-*rpoS*(RBS)-M_7_-His | *rpoS*(RBS)-M_7_ fused to His on pRKaraRed; TET^r^ | | This study | |
| Primer | Sequence 5’-3’ | Function | Source |  |
| *rpoS*-UP-F | CCGAGCTCCGACACCCTGTATTCCATTGCCTTC | For deletion *of rpoS* | This study | |
| *rpoS*-UP-R | CG GGATCC GTCGTTATCCCTTGCATGAGTTC |  |  |  |
| *rpoS*-Down-F | CG GGATCC CGGAAAACCTTAGACCCACTG |  | This study | |
| *rpoS*-Down-R | CCG CTGCAGACCTTCGTCGTCACCGACAAC |  |  |  |
| *rpoS*-com-up-F | GGGGTACCACCTGCCAAGCCCGCACCG | For complementa-tion of *rpoS-*His | This study | |
| *rpoS-*com*-*down*-* His | CCAAGCTTTCAGTGGTGGTGGTGGTGGTGCTGG  AACAGCGCGTCACTC |  | This study | |
| *rpoS-*com*-*M_3_-UR | CCCTTCTTTTTTCAGTGCCATGTCGTTATCCC |  | This study | |
| *rpoS-*com*-*M_3_-DF | GGGATAACGACATGGCACTGAAAAAAGAAGGG |  | This study | |
| *rpoS-*com*-*M_4_-UR | CAAACTCCGGCCCTTCTTTCTTGAGTGCCATGT  CGTTATC |  | This study | |
| *rpoS-*com*-*M_4_-DF | GATAACGACATGGCACTCAAGAAAGAAGGGCCG  GAGTTTG |  | This study | |
| *rpoS-*com*-*M_5_-UR | CAAACTCCGGCCCTTCCTTTTTGAGTGCCATGT  CG |  | This study | |
| *rpoS-*com*-*M5-DF | CGACATGGCACTCAAAAAGGAAGGGCCGGAGTT  TG |  | This study | |
| *rpoS*-His-UP-F | GGGGTACC TCCAAAAGCCACCACTTCCTTC | For insertion His tag into the chromosome | This study | |
| *rpoS*-His-UP-R | CGCCCGGGGGTTTTCCGTCAGTGGTGGTGGTGG  TGGTGCTGGAACAG |  | This study | |
| *rpoS*-His-Down-F | GCGCTGTTCCAGCACCACCACCACCACCACTGA  CGGAAAAC |  | This study | |
| *rpoS*-His-Down-R | GGGTCGACGCCGATGCAGAGCGACCTGTTCGC |  | This study | |
| P_BAD_-*rpoS*_M2-7_-F | CGCTCGAGTCGAACTCATGCAAGGGATAACGAC  ATGGCCCTGAAGAAGGAGGGCCCGGAGTTTGAC  CACGAT | RpoS-His cloning | This study | |
| P_BAD_-*rpoS*-His-R | CCCAAGCTTTCAGTGGTGGTGGTGGTGGTGCTG  GAACAGCGCGTCACTC |  | This study | |
| P_BAD_-*rpoS*_WT_-RBS-F | CCGCTCGAGTTTAACTTTAAGAAGGAGATATAC  CATGGCACTCAAAAAAGAAGGG |  | This study | |
| P_BAD_-*rpoS*-M_2-7_-RBS-F | CGCTCGAGTTTAACTTTAAGAAGGAGATATACC  ATGGCCCTGAAGAAGGAGGGCCCGGAGTTTGACCGCAAT |  | This study | |
| P_BAD_-*rpoS*-M_2_-RBS-F | CGCTCGAGTTTAACTTTAAGAAGGAGATATACCGG  ATCCCTCAAAAAAGAAGGG |  | This study | |
| P_BAD_-*rpoS*-M_3_-RBS-F | CGCTCGAGTTTAACTTTAAGAAGGAGATATACC  ATGGCACTGAAAAAAGAAGGG |  | This study | |
| P_BAD_-*rpoS*-M_4_-RBS-F | CGCTCGAGTTTAACTTTAAGAAGGAGATATACC  ATGGCACTCAAGAAAGAAGGG |  | This study | |
| P_BAD_-*rpoS*-M_5_-RBS-F | CGCTCGAGTTTAACTTTAAGAAGGAGATATACC  ATGGCACTCAAAAAGGAAGGGCCGGAGTTTGAC  CACGAT |  | This study | |
| P_BAD_-*rpoS*-M_6_-RBS-F | CCGCTCGAGTTTAACTTTAAGAAGGAGATATAC  CATGGCACTCAAAAAAGAGGGGCCGGAGTTTGA  CCACGAT |  | This study | |
| P_BAD_-*rpoS*-M_7_-RBS-F | CGCTCGAGTTTAACTTTAAGAAGGAGATATAC  CATGGCACTCAAAAAAGAAGGCCCGGAGTTTGA  CCACGAT |  | This study | |
| P_BAD_-*rpoS*-M_345_-RBS-F | CGCTCGAGTTTAACTTTAAGAAGGAGATATACC  ATGGCACTGAAGAAGGAAGGGCCGGAGTTTGAC  CACGAT |  | This study | |
| q*ampC*F | GTGATGAAGGCCAATGACA | RT-PCR | This study | |
| q*ampC*R | ATAGCTGAAGTAATGCGGTTC | RT-PCR | This study | |
| q*arcA*F | TCGAAGTGGTCGCCGAATC | RT-PCR | This study | |
| q*arcA*R | TGTTACCGTCGTCCCATTGC | RT-PCR | This study | |
| q*argR*F | CAAGGTCATCGCCACCAAC | RT-PCR | This study | |
| q*argR*R | CAGCGCCAACAGCAGGTC | RT-PCR | This study | |
| q*vfr*F | GTTCGTGCCAAGGTGGAATG | RT-PCR | This study | |
| q*vfr*R | TGGCTGCCGAGGGTGTAGA | RT-PCR | This study | |
| q*glnK*F | GGTGAAGATCGACGTGGC | RT-PCR | This study | |
| q*glnK*R | CACCACGAAGATCTTGCCG | RT-PCR | This study | |
| q*ihfA*F | ATCCGCCAGGCGCTGGAGC | RT-PCR | This study | |
| q*ihfA*R | GATCGGGATTTCTTCACCGG | RT-PCR | This study | |
| q*rpoS*F | GGACTCGGACAAGACCCTG | RT-PCR | This study | |
| q*rpoS*R | GCCACTGGTCGATGCTTT | RT-PCR | This study | |
| q*groES*F | GAACCGCGGTGAAGTGG | RT-PCR | This study | |
| q*groES*R | TTGCTGCCGGAGTAAGGCC | RT-PCR | This study | |
| q*lon*F | CGCTGGAGAACTACCTCGG | RT-PCR | This study | |
| q*lon*R | CCTCGATGGTGAGCAGCTC | RT-PCR | This study | |
| q*recA*F | TGAAGTTCTACGCCTCGGTC | RT-PCR | This study | |
| q*recA*R | GAAACCTTGTTCTTCACCAC | RT-PCR | This study | |
| q*recB*F | CGGTGCAGTACCGGATCTTC | RT-PCR | This study | |
| q*recB*R | GGAAGGCGTAGATCGCCTG | RT-PCR | This study | |
| q*nuoD*F | CAAGCGCCTCGACGAGTAC | RT-PCR | This study | |
| q*nuoD*R | CCACTCGAGGGCTTCCTTG | RT-PCR | This study | |
| q*nuoH*F | AGTTCTTCGGCTTCTGCACC | RT-PCR | This study | |
| q*nuoH*R | TCGATGTGGTAGCCGTCCG | RT-PCR | This study | |
| q*nuoJ*F | GCGCCATCATGGTTCTGTTC | RT-PCR | This study | |
| q*nuoJ*R | GAAGGCCCGACCCAGATG | RT-PCR | This study | |
| q*nuoK*F | GAAGTGATGATGAACGCCGC | RT-PCR | This study | |
| q*nuoK*R | GAGGCTCAGCACCAGGATG | RT-PCR | This study | |
| q*nuoL*F | GCAGACCGATATCAAGCGC | RT-PCR | This study | |
| q*nuoL*R | CATCAGGTGGAAGATCGCCG | RT-PCR | This study | |
| q*hemL*F | GGCAAGCGCGAGATCATG | RT-PCR | This study | |
| q*hemL*R | CTGATCAGGCGCAGGGTG | RT-PCR | This study | |
| q*pgi*F | GGCGCCTACAACATGGAC | RT-PCR | This study | |
| q*pgi*R | GTTCGCGCCCCAGAAGTC | RT-PCR | This study | |
| q*ubiH*F | TTGCGCGCTGATCTGGAC | RT-PCR | This study | |
| q*ubiH*R | CGAGGCGGTAGCCGAAGG | RT-PCR | This study | |

[1] Choi KH, Schweizer HP. Mini-Tn7 insertion in bacteria with single attTn7 sites: example *Pseudomonas aeruginosa*. Nat Protoc. 2006;1[1]:153-161. https://doi.org/10.1038/nprot.2006.24

[2] Liang R, Liu J. Scarless and sequential gene modification in *Pseudomonas* using PCR product flanked by short homology regions. BMC Microbiol. 2010;10:209. Published 2010 Aug 3. https://doi.org/10.1186/1471-2180-10-209

**Table S2 Concentrations (mg/L) of indicated antibiotics in the *in vitro* passaging experiment of PA14.**

|  | Single drug | | | C:A=1:1* | | | C:A=1:3* | | | C:A=1:6* | | | C:A=1:8* | | |
| --- | --- | --- | --- | --- | --- | --- | --- | --- | --- | --- | --- | --- | --- | --- | --- |
|  | Parallel 1 | Parallel 2 | Parallel 3 | Parallel 1 | Parallel 2 | Parallel 3 | Parallel 1 | Parallel 2 | Parallel 3 | Parallel 1 | Parallel 2 | Parallel 3 | Parallel 1 | Parallel 2 | Parallel 3 |
| Day1 | 0.5,1,2 | 0.5,1,2 | 0.5,1,2 | 0.5,1,2 | 0.5,1,2 | 0.5,1,2 | 0.5,1,2 | 0.5,1,2 | 0.5,1,2 | 0.25,0.5,1 | 0.25,0.5,1 | 0.25,0.5,1 | 0.25,0.5,1 | 0.25,0.5,1 | 0.25,0.5,1 |
| Day2 | 1,2,4 | 1,2,4 | 1,2,4 | 0.5,1,2 | 0.5,1,2 | 0.5,1,2 | 0.5,1,2 | 0.5,1,2 | 0.5,1,2 | 0.25,0.5,1 | 0.25,0.5,1 | 0.25,0.5,1 | 0.5,1,2 | 0.5,1,2 | 0.5,1,2 |
| Day3 | 1,2,4 | 1,2,4 | 1,2,4 | 1,2,4 | 1,2,4 | 1,2,4 | 1,2,4 | 1,2,4 | 1,2,4 | 0.5,1,2 | 0.5,1,2 | 0.5,1,2 | 0.5,1,2 | 0.5,1,2 | 0.5,1,2 |
| Day4 | 1,2,4 | 1,2,4 | 1,2,4 | 1,2,4 | 1,2,4 | 1,2,4 | 1,2,4 | 1,2,4 | 1,2,4 | 0.25,0.5.1 | 0.25,0.5.1 | 0.25,0.5.1 | 1,2,4 | 1,2,4 | 1,2,4 |
| Day5 | 4,8,16 | 4,8,16 | 4,8,16 | 2,4,8 | 2,4,8 | 2,4,8 | 0.5,1,2 | 0.5,1,2 | 0.5,1,2 | 0.25,0.5.1 | 0.25,0.5.1 | 0.25,0.5.1 | 1,2,4 | 0.5,1,2 | 0.5,1,2 |
| Day6 | 4,8,16 | 4,8,16 | 4,8,16 | 2,4,8 | 2,4,8 | 2,4,8 | 1,2,4 | 1,2,4 | 1,2,4 | 0.25,0.5.1 | 0.25,0.5.1 | 0.25,0.5.1 | 1,2,4 | 0.5,1,2 | 0.5,1,2 |
| Day7 | 4,8,16 | 4,8,16 | 4,8,16 | 2,4,8 | 2,4,8 | 2,4,8 | 2,4,8 | 1,2,4 | 0.5,1,2 | 0.25,0.5.1 | 0.25,0.5.1 | 0.25,0.5.1 | 0.5,1,2 | 0.5,1,2 | 0.5,1,2 |
| Day8 | 16,32,64 | 16,32,64 | 16,32,64 | 4,8,16 | 4,8,16 | 4,8,16 | 2,4,8 | 1,2,4 | 1,2,4 | 0.25,0.5.1 | 0.25,0.5.1 | 0.25,0.5.1 | 1,2,4 | 1,2,4 | 1,2,4 |
| Day9 | 32,64,128 | 32,64,128 | 32,64,128 | 8,16 | 4,8,16 | 4,8,16 | 4,8,16 | 2,4,8 | 2,4,8 | 0.25,0.5.1 | 0.25,0.5.1 | 0.25,0.5.1 | 0.5,1,2 | 0.5,1,2 | 0.5,1,2 |
| Day10 | 32,64,128 | 32,64,128 | 32,64,128 | 4,8,16 | 4,8,16 | 4,8,16 | 4,8,16 | 4,8,16 | 4,8,16 | 0.5,1,2 | 0.5,1,2 | 0.5,1,2 | 0.5,1,2 | 0.5,1,2 | 0.5,1,2 |

* Represents only the ceftazidime concentration. The concentration of azithromycin is inferred based on the proportional relationship with ceftazidime/avibactam (CZA). The concentration of avibactam was fixed at 4 mg/L. C, CZA; A, azithromycin.

**Table S3 Concentrations (mg/L) of indicated antibiotics in the *in vitro* passaging experiment of PA14.**

|  | CZA+FOS* | CZA+AMK* | CZA+CIP* | CZA+ATM* | CZA+AZM* |
| --- | --- | --- | --- | --- | --- |
| Day1 | 0.25,0.5,1 | 0.25,0.5,1 | 0.25,0.5,1 | 0.25,0.5,1 | 0.25,0.5,1 |
| Day2 | 0.5,1,2 | 0.5,1,2 | 0.5,1,2 | 0.5,1,2 | 0.5,1,2 |
| Day3 | 1,2,4 | 0.5,1,2,4 | 0.5,1,2,4 | 0.5,1,2,4 | 1,2,4 |
| Day4 | 2,4,8 | 0.5,1,2,4 | 0.5,1,2,4 | 2,4,8 | 1,2,4 |
| Day5 | 4,8,16 | 1,2,4 | 0.5,1,2 | 4,8,16 | 1,2,4 |
| Day6 | 4,8,16 | 1,2,4 | 0.5,1,2 | 4,8,16 | 1,2,4 |
| Day7 | 2,4,8 | 2,4,8 | 1,2,4 | 8,16,32 | 2,4,8 |
| Day8 | 4,8,16 | 2,4,8 | 0.5,1,2 | 8,16,32 | 8,16,32 |

**Table S4 Concentrations (mg/L) of indicated antibiotics in the *in vitro* passaging experiment of CI-PA41.**

|  | CZA+FOS* | CZA+AMK* | CZA+CIP* | CZA+ATM* | CZA+AZM* |
| --- | --- | --- | --- | --- | --- |
| Day1 | 0.25,0.5,1 | 0.25,0.5,1 | 0.25,0.5,1 | 0.25,0.5,1 | 0.25,0.5,1 |
| Day2 | 0.5,1,2 | 0.5,1,2 | 0.5,1,2 | 0.5,1,2 | 0.5,1,2 |
| Day3 | 1,2,4 | 0.5,1,2,4 | 0.5,1,2,4 | 0.5,1,2,4 | 1,2,4 |
| Day4 | 2,4,8 | 0.5,1,2,4 | 2,4,8 | 2,4,8 | 1,2,4 |
| Day5 | 4,8,16 | 1,2,4 | 2,4,8 | 2,4,8,16 | 0.5,1,2,4 |
| Day6 | 4,8,16 | 1,2,4 | 2,4,8 | 2,4,8,16 | 1,2,4 |
| Day7 | 8,16,32 | 2,4,8 | 4,8,16 | 8,16,32 | 2,4,8 |
| Day8 | 4,8,16 | 2,4,8 | 4,8,16 | 8,16,32 | 8,16,32 |

* Represents only the concentration of ceftazidime. Concentrations of other antibiotics are inferred based on the proportional relationship with ceftazidime/avibactam. The concentration of avibactam was fixed at 4 mg/L. FOS: fosfomycin; AMK: amikacin; CIP: ciprofloxacin; ATM: aztreonam; AZM: azithromycin.

**Table S6 The relative mRNA expression levels of genes associated with mutation promotion under antibiotic treatment in PA14.**

| **Gene** | **CZA** | | **AZM** | | **C+A** | |
| --- | --- | --- | --- | --- | --- | --- |
|  | Average | SD | Average | SD | Average | SD |
| *arcA* | 1.145 | 0.134 | 0.030 | 0.000 | 0.465* | 0.078 |
| *argR* | 1.060 | 0.071 | 0.460 | 0.071 | 0.525* | 0.092 |
| *vfr* | 0.915 | 0.049 | 0.195 | 0.021 | 0.395* | 0.064 |
| *glnK* | 1.150 | 0.552 | 0.290 | 0.014 | 0.765 | 0.120 |
| *ihfA* | 0.810 | 0.071 | 0.185 | 0.007 | 0.660* | 0.085 |
| *rpoS* | 1.362 | 0.021 | 0.359 | 0.014 | 0.370** | 0.009 |
| *groES* | 1.045 | 0.049 | 0.165 | 0.007 | 0.180* | 0.014 |
| *lon* | 0.930 | 0.028 | 0.360 | 0.057 | 0.440** | 0.028 |
| *recA* | 1.115 | 0.120 | 0.880 | 0.141 | 0.640* | 0.028 |
| *recB* | 0.975 | 0.092 | 0.820 | 0.071 | 0.570 | 0.127 |
| *nuoD* | 0.960 | 0.057 | 0.520 | 0.071 | 0.485* | 0.092 |
| *nuoH* | 1.050 | 0.184 | 0.495 | 0.092 | 0.835 | 0.134 |
| *nuoJ* | 0.915 | 0.049 | 0.545 | 0.191 | 0.460* | 0.099 |
| *nuoK* | 0.885 | 0.064 | 0.330 | 0.071 | 0.445* | 0.092 |
| *nuoL* | 0.980 | 0.141 | 0.460 | 0.057 | 0.385* | 0.078 |
| *hemL* | 0.875 | 0.078 | 0.635 | 0.021 | 0.680 | 0.071 |
| *pgi* | 0.855 | 0.049 | 0.510 | 0.085 | 0.475* | 0.078 |
| *ubiH* | 1.025 | 0.049 | 0.320 | 0.028 | 0.330** | 0.028 |

SD: Standard Deviation**;** Significant differences between C+A and CZA treatment groups were determined using Student’s t test. ***,** P<0.05, **, P<0.01.

**Table S8 Antimicrobial susceptibility (MIC, mg/L) of wild type PA14 and clinical isolates to clinical antibiotics.**

|  | MEM | IPM | CAZ | CAZ/AVI | AZM |
| --- | --- | --- | --- | --- | --- |
| PA14 | 0.25 | 0.25 | 2 | 2 | 64 |
| CI-PA-41 | >64 | >64 | 32 | 4 | 128 |
| CI-PA-18 | >64 | >64 | 32 | 4 | 128 |
| CI-PA-36 | >64 | >64 | 32 | 4 | >128 |
| CI-PA-37 | >64 | >64 | 32 | 4 | >128 |
| CI-PA-88 | >64 | >64 | 32 | 4 | 128 |
| CI-PA-103 | >64 | >64 | 64 | 4 | 128 |
| CI-PA-105 | >64 | >64 | 64 | 4 | 128 |
| CI-PA-106 | >64 | >64 | 64 | 4 | 128 |
| CI-PA-111 | >64 | >64 | 32 | 4 | >128 |
| CI-PA-135 | 8 | 16 | 2 | 2 | 128 |
| CI-PA-145 | 16 | 16 | 2 | 1 | 128 |

MEM: Meropenem; IPM: Imipenem; CAZ: Ceftazidime; AVI: Avibactam; AZM: Azithromycin
